# Laboratory dielectric measurements to evaluate the conductivity change in the presence of chelating agent with different brines

**DOI:** 10.1038/s41598-022-23964-6

**Published:** 2022-11-18

**Authors:** Sulaiman A. Alarifi, Mohamed Mahmoud

**Affiliations:** Department of Petroleum Engineering, King Fahd University of Petroleum & Minerals (KFUPM), Dhahran, 31261 Saudi Arabia

**Keywords:** Fossil fuels, Natural gas

## Abstract

In the oil and gas reservoirs, the interaction between the injected fluids and the reservoir fluids and rocks plays a major role in the productivity of any oil and gas field. Studying the ion exchange between reservoir fluids and the injected fluids for water flooding or chemical enhanced oil recovery purposes would help in optimizing the oil displacement process and hence the productivity form such secondary or tertiary recovery mechanisms. Chelating agents are used for enhance oil recovery to improve the oil displacement and sweep efficiency by altering the reservoir rock’s surface. When it comes to fluid-rock interaction, conductivity and ionic activity of the injected water will have a great impact on the rock’s surface charge and therefore in the reservoir’s wettability. Dielectric laboratory measurements have the ability to observe the change in conductivity at high frequency due to the presence of free ions and salts in fluids. In this work, observing the effect of chelating agent with different salts on high frequency conductivity using laboratory dielectric measurements has been conducted. Introducing laboratory dielectric measurement could be a valuable tool in the lab as an evaluation technique into the ion exchange that occurs between different fluids from the reservoir with different brines and additives to study the fluid–fluid interaction activities. It can be also utilized to investigate the maximum chelating capacity of different chelating agents with different cations which can be reflected by the change in conductivity.

## Introduction

Dielectric logging introduced since the 1970s, it was made to measure the water porosity in flushed zone. The dielectrolog tool has been used in the field for interpreting water saturation of formation and watered-out zones^1^. It eventually disappeared due to modern accuracy of other devices. New enhanced dielectric measurement logging tools are introduced and tested. The new tools are available and being used nowadays in many fields and they are more capable to adopt for many reservoir conditions. Dielectric tools have a great advantage and better accuracy over other tools especially when the salinities of connate water and injected water are varying widely. The main idea behind this tool is the dielectric permittivity and conductivity that controls the electromagnetic wave propagation in pore space. Taking into account that water has a high dielectric permittivity that is much higher than any other fluid or mineral in the reservoir; dielectric measurements become very sensitive to any water presence at the rock pore scale^2^.

Basically, dielectric permittivity of a material is a measure of a material’s ability to store charge when an electric field is applied^3^. In petroleum industry, dielectric permittivity measurements can be used to distinguish between water and oil saturated zones because of the large contrast between relative dielectric permittivity of oil and water. For water it is around 80 and oil around 2^4,5^. The newly developed tools for dielectric measurements are performed at different discrete frequencies from 20 MHz to 1 GHz; main advantage is the continuous measurement of dielectric dispersion along the reservoir layers^6,7^. In the literature, many have related dielectric measurements to other properties; such as water saturation, water salinity and pore structure using several models and interpretations.

Water has a high dielectric permittivity that is much higher than any other fluid or mineral in the reservoir as shown in Table 1. Dielectric measurements become very sensitive to any water presence at the rock pore scale. Electronic polarization is a dominate source of dielectric behavior for most matrix constituents such as quartz, calcite and dolomite. Water molecules are polar because of its shape, due to the nonsymmetrical arrangements of atoms. Therefore, it acts like a permanent electric dipole. Dielectric permittivity of a material is a measure of the ability of a material to store charge when an electric field is applied^3^ which is called the dielectric permittivity of free-space resulting in a dimensionless quantity known as the relative permittivity (Ɛ_r_).

**Table 1:** Relative dielectric permittivity (Ɛ_r_) of fluids and minerals^2^.

Material	Ɛ_r_
Quartz	4.4
Sandstone	4.65
Limestone	7.59.2
Dolomite	6.8
Clay	5.05.8
Anhydrite	6.4
Halite	5.9
Gypsum	4.16
Oil	2–2.2
Gas (Air)	1.0
Water*	5078

*Salinity increase will reduce permittivity of water due to several mechanisms.

Water tends to occupy most of the rock surface and the small pores when injected into a water-wet reservoir^8^. This water will form a continuous phase, while oil will be isolated in pockets even at high oil saturations. On the contrary, oil-wet surfaces will be in contact with oil as its preferred phase and water will be isolated. Generating an electric field on the water-wet rocks, the dissolved ions in water will have the capability to move along the film of water surrounding and occupying the cores which makes water travel for long distances before being trapped. While, if the electric field is generated on oil-wet rocks, the distance traveled by the ions contained in spherical water droplets inside the larger pores, will have more or less water inclusions creating two different electrical responses and allowing determination of rock wettability^9^. Basically, permittivity considered being the sensitivity of a medium to an electric field excitation. Main physical phenomena contribute to the permittivity: Displacement of the electronic cloud of atoms, the coherent orientation on pre-existing microscopic electric dipoles and the polarization effect at the interfaces. Sources of these three mechanisms are: Electronic polarization (rock permittivity), Molecular orientation (water molecules) and interfacial polarization (pore geometry and ions) known as Maxwell–Wagner effect. The term polarization implies the orientation in the direction of the applied external field ($\overrightarrow{\mathrm{E}}$). The effectiveness of a given polarization process varies with the frequency and the molecule/particle involved^7^. According to Xiuwen et al. dielectric constants which are presented as the relative permittivity Ɛ_r_ for crude oil and minerals of sedimentary rocks are small (2–8), for water very high (80) it depends mainly on water volume. With 60 MHz frequency used, experimental formula for dielectric constant for the field of investigation for sandstone depends on water saturation, porosity, shaliness (in volume percent) have been introduced. Experiments showed that dielectric constant decreases as salinity increase. However, rocks saturated with water with salinity less than 20,000 ppm have little change in dielectric constant^10^. Even that there are many dielectric forward models exist in the literature to convert dielectric measurements into water saturation, water salinity, rock texture, no model to convert it to wettability.

Several studies in the literature relied on taking the dielectric measurements for the saturated rock samples at different wettability conditions. Then, the behavior of these dielectric measurements in response to different wettability conditions is studied and analyzed. A study conducted by Bona et al. included 8 samples of Berea sandstone and 16 sintered glass filters, with two different mesh sizes at different wettability conditions. The multi-frequency dielectric measurement on fully brine saturated core samples showed how wettability strongly affects the dielectric properties of the samples investigated. Water-wet samples showed higher permittivity values and dispersivity compared to oil-wet samples which attributed to Maxwell–Wagner effect^11^. Other authors studied and developed effective-theory based models and methods to predict rocks wettability through using systematic laboratory assessment of water-wet carbonate and clastic rocks altered to oil-wet rocks and all agree that more oil-wet rocks have lower Maxwell–Wagner effect and lower dielectric constant at MHz frequency range^11–14^. Garcia and Heidari have characterized the multi-frequency dielectric dispersion in mixed wet rocks. They successfully built an analytical model to simulate the results obtained for 6 sand stone samples which were used in Bona et al.’s (2001) study. They utilized Maxwell Garnet and Hanai-Bruggeman’s models to calculate the effective permittivity by considering the oil and water saturations. In their approach, they introduced two metrics to characterize wettability; fraction of grains which are water-wet and inner layer and oil layer thickness which coats the grains. Their findings matched Bona et al.’s (2001) outcomes and showed how water-wet grains fraction drops for oil-wet samples^13^. Al-Ofi et al. conducted laboratory core measurements on different core samples which their wettability state has been altered and obtained USBM wettability index at each step. Their findings showed the water depolarization factor as obtained from Maxwell Garnet’s model correlates well with wettability condition of the samples and drops from 1.40 for the initial water-wet sample to 0.36 for strongly oil-wet sample which resamples the water geometry tends to be more spherical shaped inside the pore system at oil-wet state^12^.

In a study by Beloborodov et al.^15^ they concluded that at high frequencies (above 10 MHz) real relative permittivity will show a different linear trend with porosity in fresh water and brine saturated samples. The showed that salinity and cation composition of the pore fluid have a negligible effect on dielectric measurements at high frequency. Shcherbakov et al.^16^ stated that the specific electrical conductivity of aqueous solutions of inorganic metal salts increases proportionally with the limiting high frequency electrical conductivity of the solvent with increasing temperature. Ishtaiwi et al.^17^ studied the electrical properties of Dead Sea water at 200 MHz to 9 GHz frequency range. They concluded that Dead Sea water can be used efficiently in low-frequency applications that require a high dielectric conductive medium as its conductivity is very high as expected due to the high salinity of around 345,000 ppm.

Dielectric measurements is utilized in several aspects and for different fields of science. In a study by Vera et al.^18^ they discussed the fundamentals of electromagnetic sensors which are based on soil dielectric permittivity. These sensors are designed to automate irrigation and optimize the utilization of water, energy and labor. Jones et al.^19^ discussed the utilization of dielectric measurements used for inferring moisture content in grains. As measuring the moisture content is crucial for the harvesting, processing, storing and marketing of cereal grains, oilseeds and legumes. They indicated that permittivity measurements in cereal grains and legumes are most significantly influenced by water content and status as the porous media permittivity measurements are highly affected by the presence of free and bound water. In a study by Ramasamy et al.^20^ they utilized dielectric measurements to identify fiber content in edible flours.

Chelating agents are organic compounds that form soluble, complex molecules with metal ions that can control the reactivity of multivalent metal ions by inactivating the ions (seize metal ions and control them) so that they cannot normally react with other elements or ions. Using chelating agents in enhanced oil recovery depends on the phenomena of capturing cations from the water injected and the formation brine which will lead for cation release from the rock in order to achieve equilibrium at the rock surface. Also, surface of the rock will change to more water wet through promotion of ion exchange whenever cations are being captured for the rock surface or from the connate water. Chelating agent fluid system could be added to seawater without dilution and a main advantage of chelating agents is the capability to use them at very low concentrations. Also, a main advantage of chelating agents is the effect they have on the rock dissolution process where they force the rock to release the oil that is attached to the surface and this leads to an increase in oil recovery^21^. They have been successfully used as an additive in the oil and gas industry in many aspects. For example, for scale removal process, iron control and matrix stimulation. Recently, they are being used as standalone fluids for enhanced oil recovery, stimulation and water alteration applications^22^.

Chelating agents proved their effect on mineralogy and surface charge by their ability to absorb certain cations which lead to more water wet rock surface^21^. Evaluating these effects on sandstone rocks along with the change in dielectric measurements is what this work is aiming to reach. In this work, the effect of certain ions, chelating agents and different water salinities on conductivity is investigated using laboratory dielectric measurements at high frequency (500 MHz to 1 GHz). The use of laboratory dielectric measurement setup as an evaluation technique, relating the effect of seawater, low salinity water, deionized (DI) water and chelating agent on certain cations with dielectric and conductivity measurements is presented to explain the fluid–fluid interaction in terms ion exchange activities^23^.

## Material and methods

**Figure 1:**
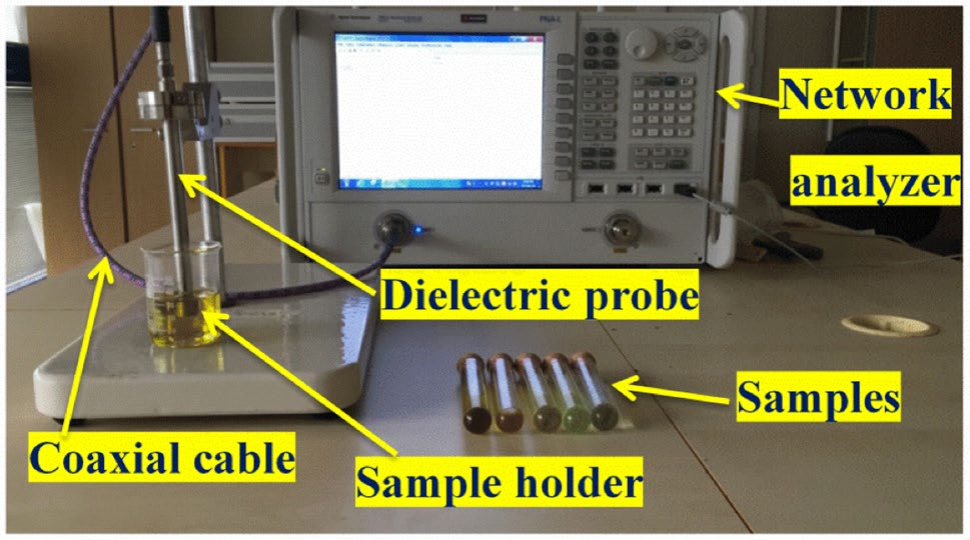
Similar experimental setup of Keysight 85070E Dielectric Probe Kit, dielectric experimental apparatus^24^.

For dielectric equipment, the type of sample holder (fixture) required depends on the physical type of the material (solid, liquid, powder, gas). Dielectric measurements are performed on fluids only with different salinities and on powders from sandstone rocks with the different water based fluids. Before testing the samples, deionized water and different water salinities are tested to as a base scenario before any additives. The device used is the Keysight 85070E Dielectric Probe Kit shown in Fig. 1 connected to a Keysight network analyzer, determines the intrinsic electromagnetic properties of many dielectric materials. The 85070E has a frequency range of 200 MHz to 50 GHz. The network analyzer is calibrated using a set of short, open (air), and deionized water. The quality of contact between the sample and the probe is essential in reducing any measurement errors. The open-ended probe operates in reflection mode. To perform a reflection measurement, only one port of the network analyzer is used. The S11 scattering parameter (S-parameter), also called reflection coefficient, is measured by the network analyzer. The S-parameters are recorded in the form of an amplitude (in dB) and phase (in degrees) as a function of frequency which was varied from 500 MHz to 20 GHz. The device yields two values: Ɛ_r_ and Ɛ^″^ as function of frequency. Ɛ_r_ is the real part of the complex permittivity representing the relative dielectric constant (unitless), relative to vacuum permittivity. Ɛ^″^ is the dielectric loss factor which is imaginary part of the complex permittivity and relates to conductive loss and to dissipation, it is also a unitless value. These two outcomes of the device readings are then inverted to estimate the conductivity and the permittivity of the sample. The quality of the measurement technique is routinely verified using the known dielectric property deionized water. The common expression for dielectric properties where they are related to complex number (complex permittivity Ɛ^*^) where it has a real and imaginary part is:

1 $$\varepsilon ^{*} = \varepsilon ^{\prime } + \frac{{{\text{i}} {\upsigma}}}{{\upomega \upvarepsilon _{0} }}$$

where Ɛ^′^ is relative dielectric constant (relative permittivity) which is a dimensionless quantity equal to the ratio of the medium and vacuum permittivities, $\mathrm{i}$ = $\sqrt{-1 }$, $\upsigma$ is conductivity in (S/m),$\upomega$ is circular frequency (2πƒ) (rd/s) , Ɛ_0_ is the vacuum permittivity in Ɛ_0_ = 8.854187817 × 10^−12^ F/m and ƒ is the frequency (1/second or Hertz). $\upsigma$ is the conductivity as a function of frequency (Siemens/meter).

2 $${\upsigma } = {{\upvarepsilon^{\prime\prime}\upomega \upvarepsilon }}_{0}$$

where Ɛ^″^ is the dielectric loss factor which is the imaginary part of the complex permittivity (unitless). All the measurements are done on ambient conditions. At least three readings are conducted showing a high consistency in the results. The average is taken of these readings and this procedure is repeated another time to insure the accuracy of the measurements. The uncertainty of the device is within 5% error. The frequency ranges of the device considered on the comparison is from 500 MHz to 20 GHz. Knowing that the dielectric logging tool has a frequency from 20 MHz to 1 GHz. Therefore, for comparison, readings are taken from 500 MHz to 1 GHz, presenting the effect of volumetric polarization which is due to the molecule volume (no coupling or interfacial polarization). The effect of presence of ions is clearly shown on the conductivity readings rather than the relative dielectric constant.

Powder samples from Berea mixed with seawater, Berea mixed with low salinity water and Bandera mixed with seawater are used here to measure the dielectric properties. Table 2 summarizes the mineralogy of the two sandstone cores. The fluids used are Arabian Gulf seawater and low salinity water –diluted 10 times- prepared by dissolving salt in deionized water. Ionic content shown in Table 3.

**Table 2:** Mineralogy of sandstone cores in wt%^26^.

Mineral	Berea	Bandera
Quartz	87	58
Dolomite	1	16
Calcite	2	–
Kaolinite	4	3
Illite	1	10
Chlorite	2	1
Potassium feldspar	3	–
Plagioclase	–	12
Molecular weight (gm)	98.94	149.47

**Table 3:** Composition of Formation water, Arabian Gulf Seawater and low salinity water (total dissolved solids).

Ions	Seawater (SW)	Low salinity water (LS)
Sodium	18,300	1830
Calcium	650	65
Magnesium	2110	211
Sulfate	4290	429
Chloride	32,200	3220
Bicarbonate	120	12
Total dissolved solids (TDS)	57,670	5767

**Figure 2:**
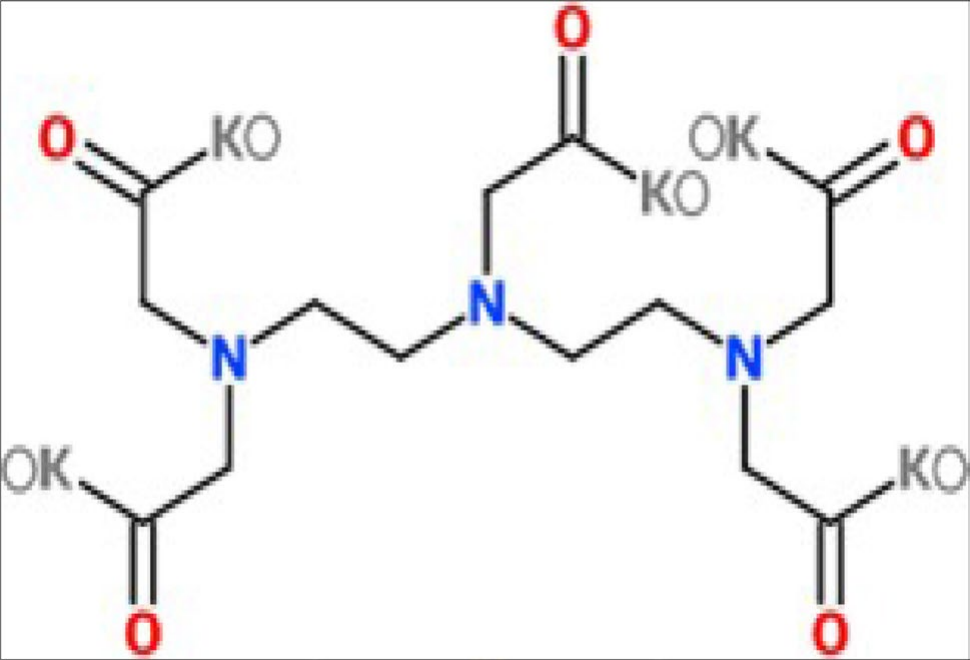
Chemical structure of DTPA-K5 Chelating Agent (After Mahmoud and Abdelgawad^25^).

A commercially available chelating agent named DTPA-K5 has been used. DTPA-K5 (diethylenetriaminepentaacetic acid, Potassium salt) chemical structure shown in Fig. 2. DTPA acid molecular weight = 393.35 (C_14_H_23_N_3_O_10_) and the molecular weight of DTPA-K5 (C_14_H_18_N_3_O_10_K_5_) = 583.8 gm with pH value of 11 and density of 1.25 g/mL^21^.

**Figure 3:**
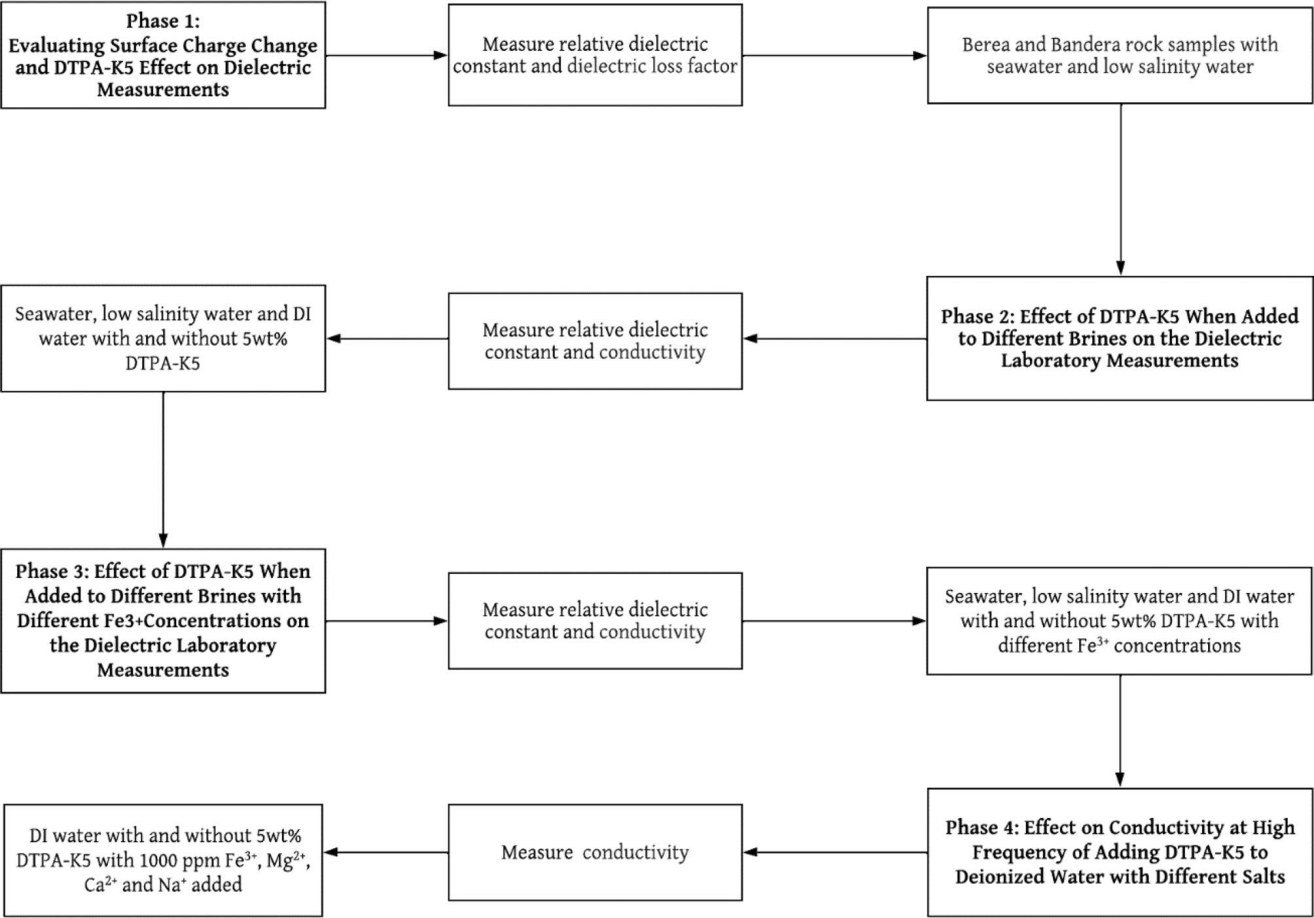
Methodology flowchart.

FeCl_3_ salt has been used to produce several Fe^3+^ cation concentrations in deionized water as shown in Table 4. The same is done to produce 1000 ppm of Ca^2+^, Na^+^ and Mg^2+^ as shown in Tables 5, 6, 7. Figure 3 is a flowchart summarizing the methodology with the four phases followed in this work.

**Table 4:** Different Fe^3+^ concentrations prepared using FeCl_3_ salt in DI water.

Fe^3+^ ion concentration (ppm)	Amount of FeCl_3_ salt (gm)	Deionized water volume (liter)	Cl^-^ ion concentration (ppm)
0	0	0.02	0
500	0.029	0.02	953.45
1000	0.058	0.02	1906.89
2000	0.116	0.02	3813.79
3000	0.174	0.02	5720.68
4000	0.233	0.02	7627.57

**Table 5:** Different Ca^2+^ concentrations prepared using CaCl_2_ salt in DI water.

Ca^2+^ ion concentration (ppm)	Amount of CaCl_2_.2H_2_O salt (gm)	Deionized water volume (liter)	Water salinity (ppm)	Cl^−^ ion concentration (ppm)
1000	0.073	0.02	2774.556	1774.56

**Table 6:** Different Na^+^ concentrations prepared using NaCl salt in DI water.

Na^+^ ion concentration (ppm)	Amount of NaCl salt (gm)	Deionized water volume (liter)	Water salinity (ppm)	Cl^−^ ion concentration (ppm)
1000	0.051	0.02	2544	1544.82

**Table 7:** Different Mg^2+^ concentrations prepared using MgCl_2_ salt in DI water.

Mg^2+^ ion concentration (ppm)	Amount of MgCl_2_.6H_2_O salt (gm)	Deionized water volume (liter)	Water salinity (ppm)	Cl^−^ ion concentration (ppm)
1000	0.167	0.02	3921.811	2921.81

## Results and discussions

### Evaluating surface charge change and DTPA-K5 effect on dielectric measurements

**Figure 4:**
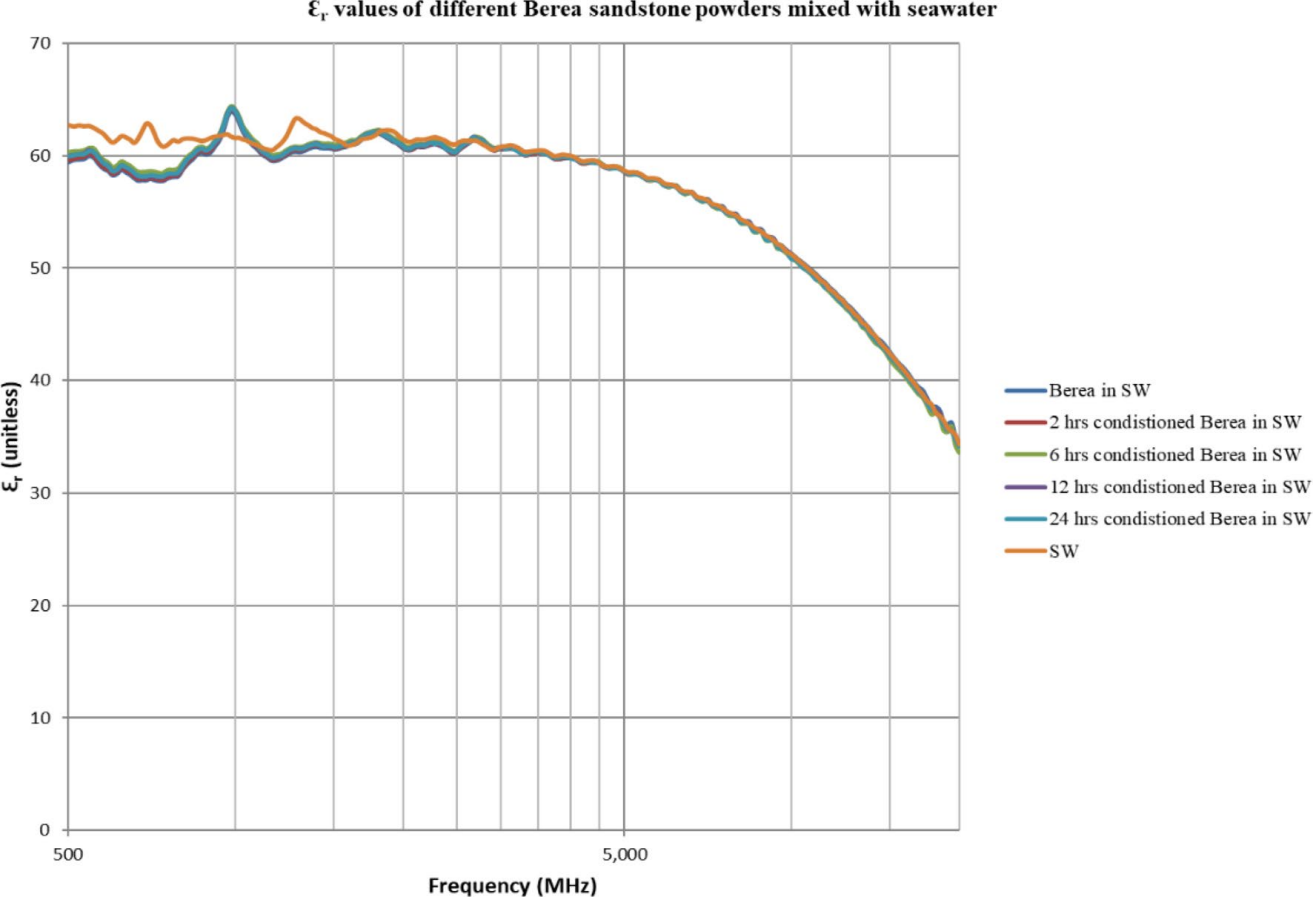
Relative dielectric constant (Ɛ_r_) values of different Berea sandstone powders mixed with seawater.

**Figure 5:**
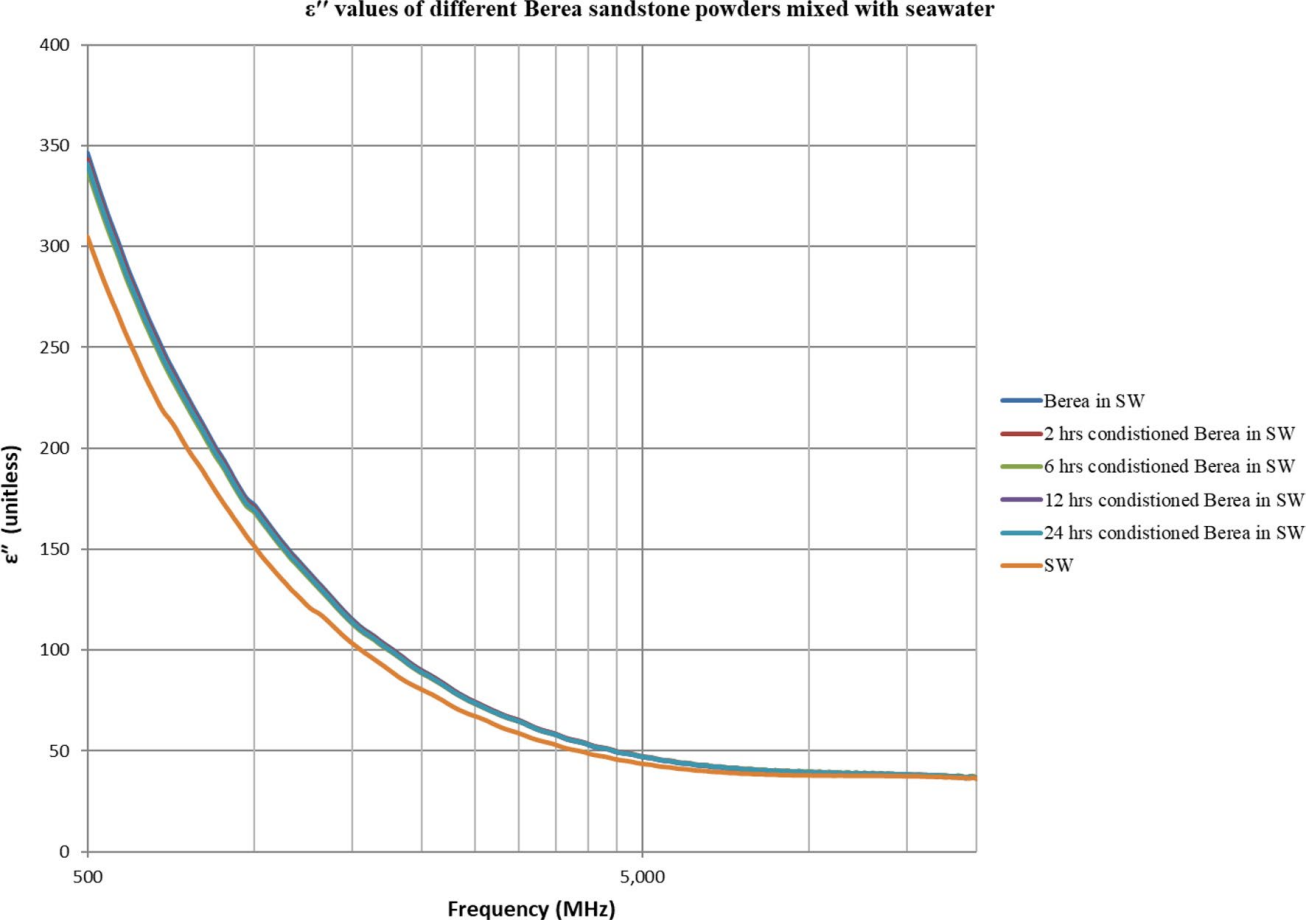
Dielectric loss factor (Ɛ^″^) values of different Berea sandstone powders mixed with seawater.

**Figure 6:**
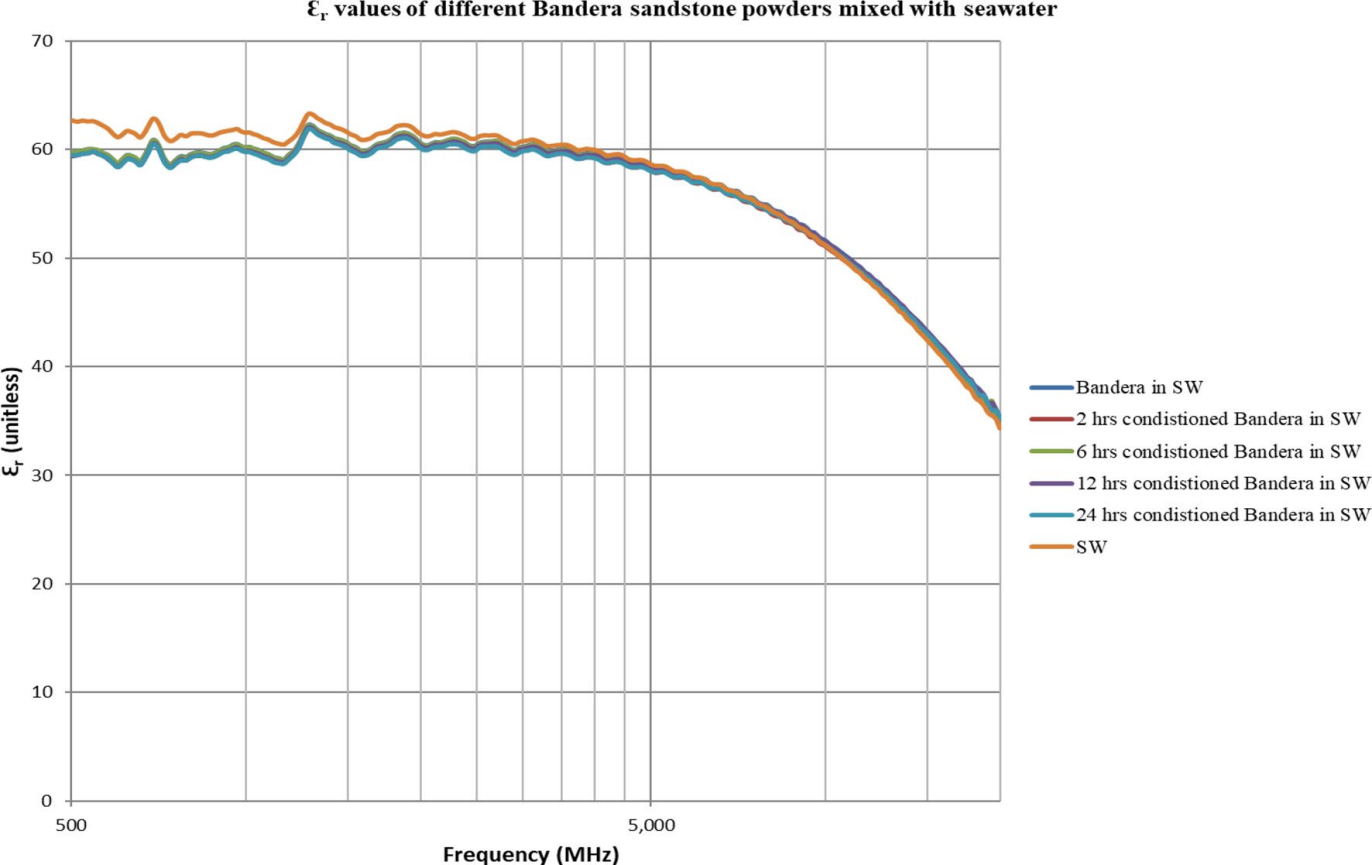
Relative dielectric constant (Ɛ_r_) values of different Bandera sandstone powders mixed with seawater.

**Figure 7:**
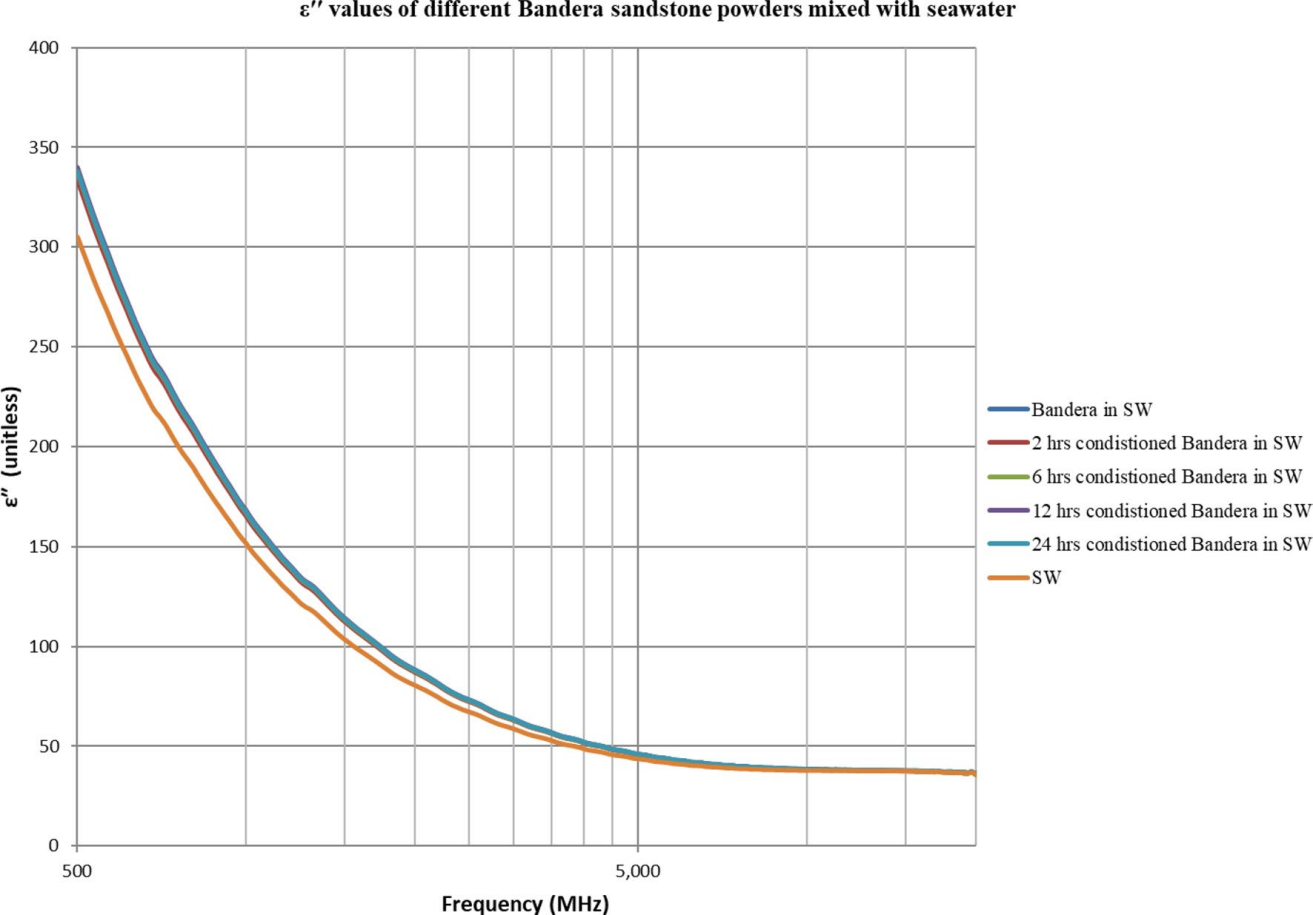
Dielectric loss factor (Ɛ^″^) values of different Bandera sandstone powders mixed with seawater.

**Figure 8:**
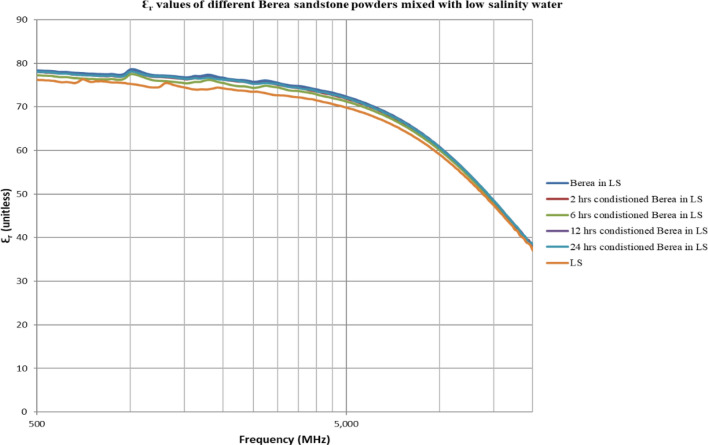
Relative dielectric constant (Ɛ_r_) values of different Berea sandstone powders mixed with low salinity water.

**Figure 9:**
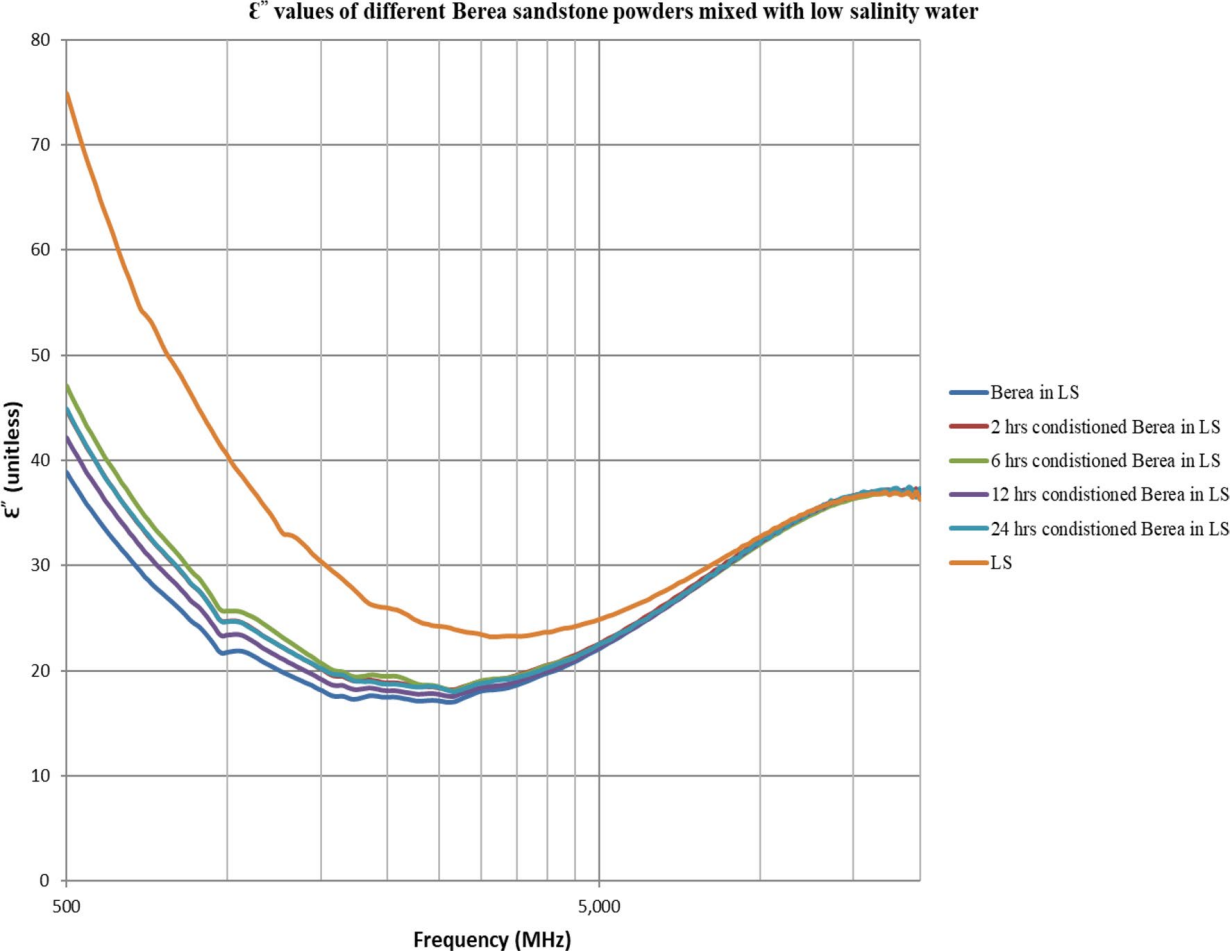
Dielectric loss factor (Ɛ^″^) values of different Berea sandstone powders mixed with low salinity water.

The double layer effect is not observed (Figs. 4, 5, 6, 7, 8, 9) using the dielectric measurement due to the high frequency (starts from 500 MHz comparing to 2 and 20 Hz in zeta potential measurements), in which double layer polarization is not paramount. Having the powder conditioned for any time period gave similar values of Ɛ_r_ and Ɛ^″^ as if it has not been conditioned at all. The difference here is within the uncertainty of the device (5%). The clear effect shown in these measurements is the change in the dielectric loss factor value of seawater or low salinity water when powder is added, indicating an increase in the conductivity by the powder particles’ presence. Dielectric loss factor value is higher for low salinity water without Berea sandstone powders, the difference is much clearer at the lower frequency range (Fig. 9). Using laboratory dielectric measurements to observe the change in the surface charge is not applicable.

### Effect of DTPA-K5 when added to different brines on the dielectric laboratory measurements

**Figure 10:**
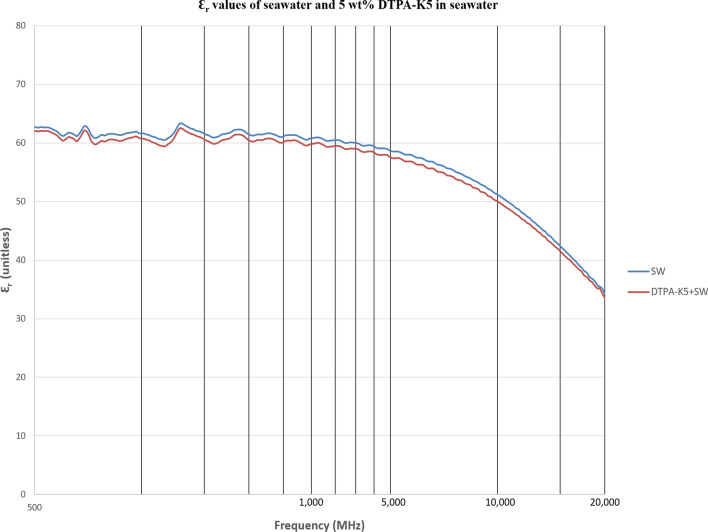
Relative dielectric constant (Ɛ_r_) values of seawater and 5 wt% DTPA-K5 in seawater.

**Figure 11:**
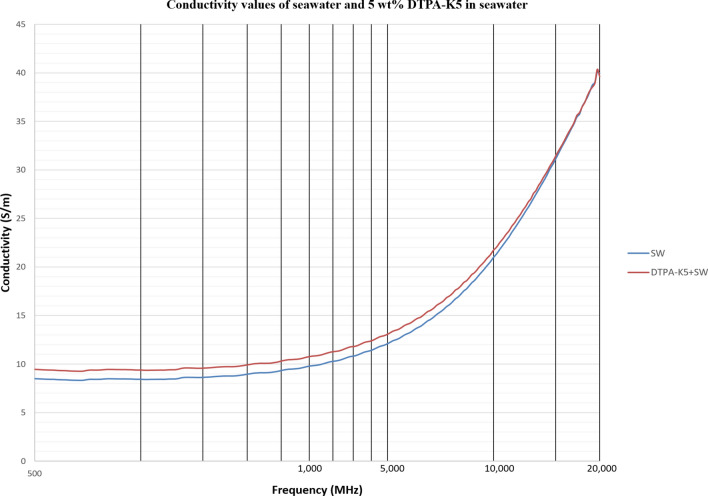
Conductivity ($\upsigma$) values of seawater and 5 wt% DTPA-K5 in seawater.

**Figure 12:**
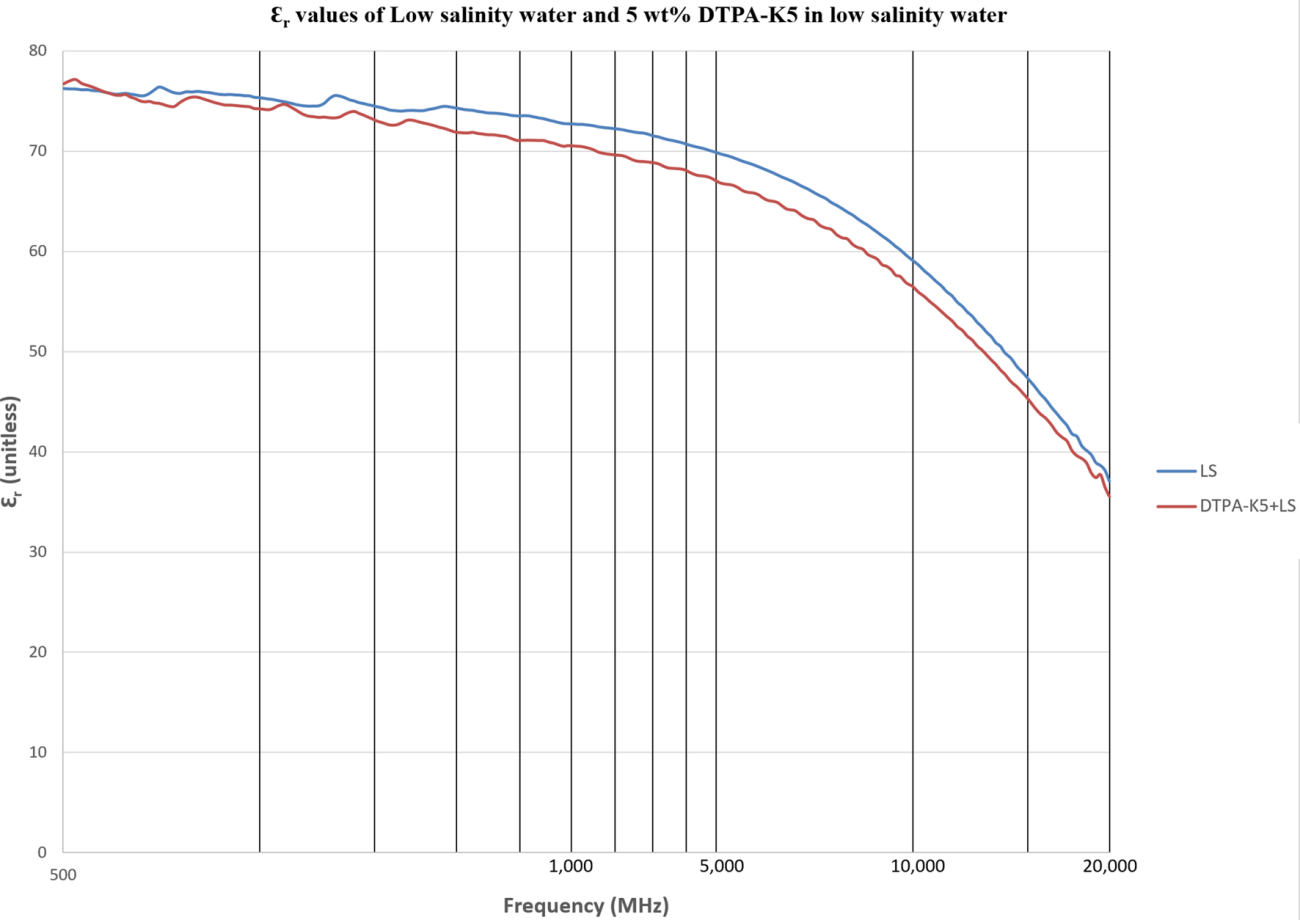
Relative dielectric constant (Ɛ_r_) values of low salinity water and 5 wt% DTPA-K5 in low salinity water.

**Figure 13:**
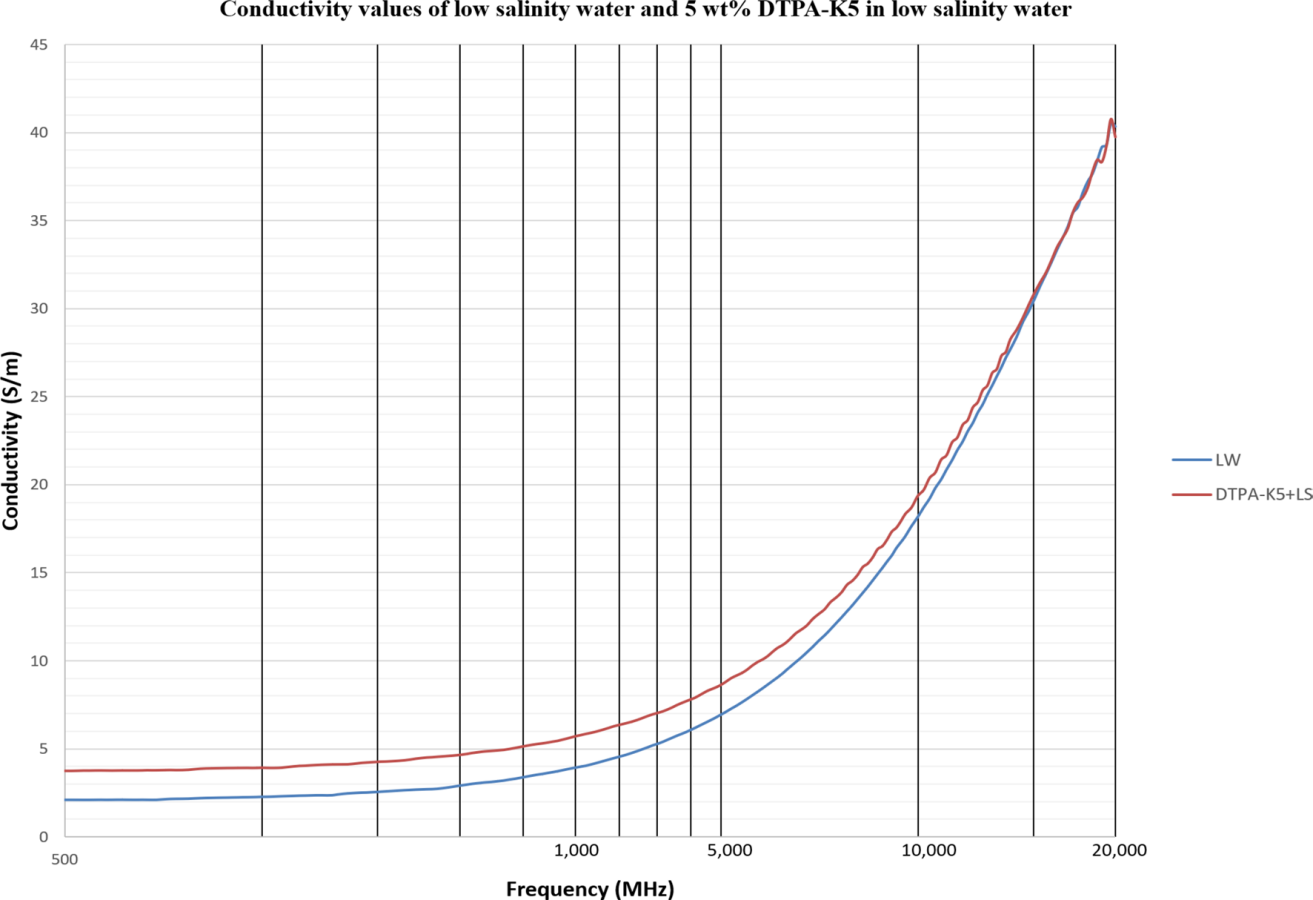
Conductivity ($\upsigma$) values of low salinity water and 5 wt% DTPA-K5 in low salinity water.

**Figure 14:**
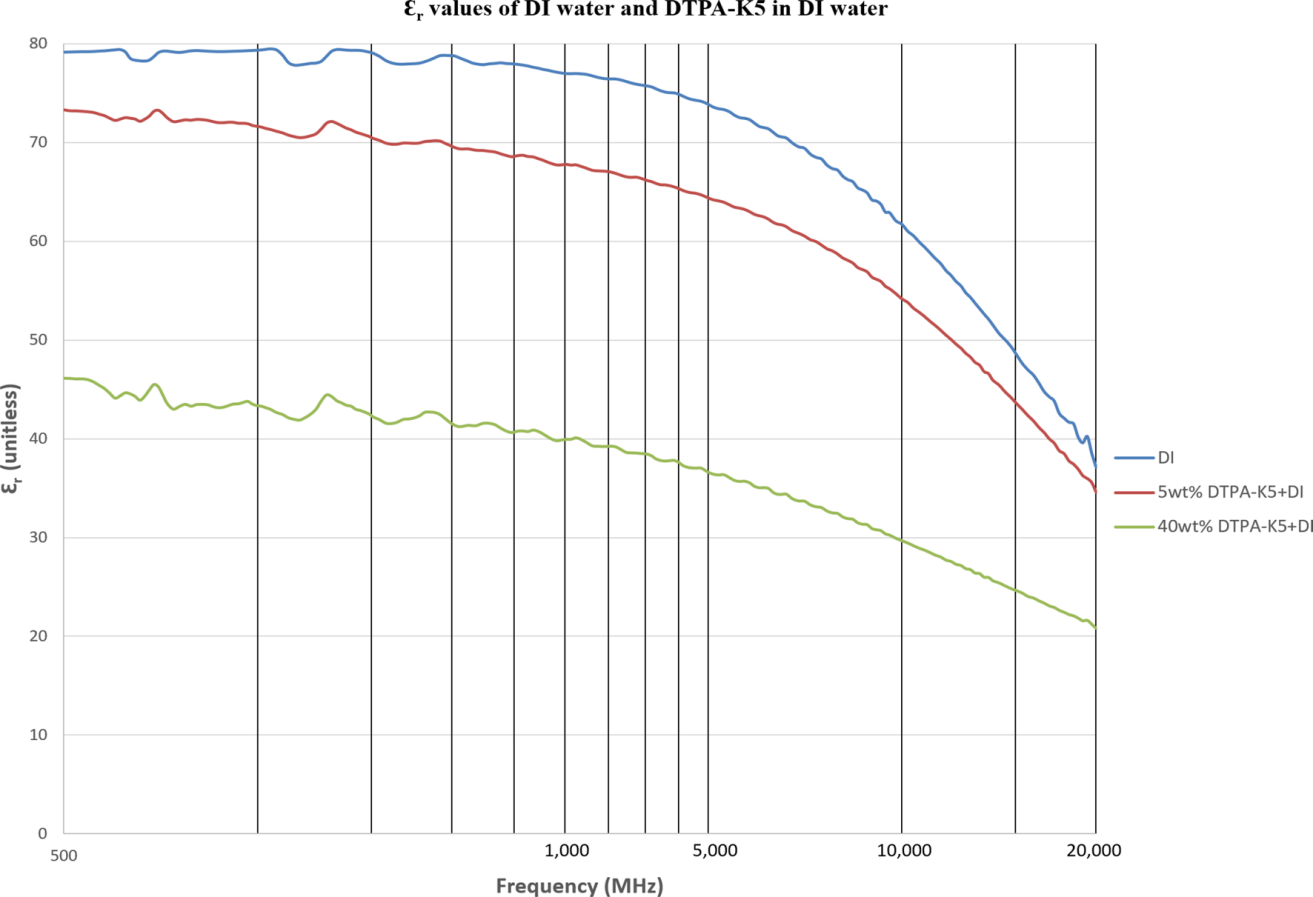
Relative dielectric constant (Ɛ_r_) values of DI water, 5 wt% DTPA-K5 in DI water and 40 wt% DTPA-K5 in DI water.

**Figure 15:**
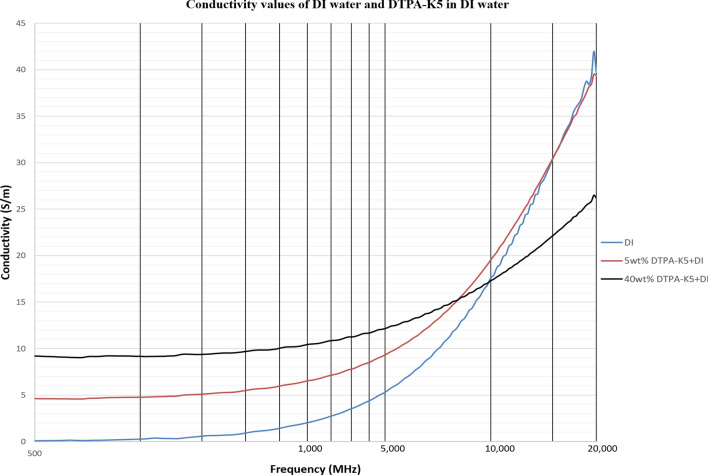
Conductivity ($\upsigma$) values of DI water, 5 wt% DTPA-K5 in DI water and 40 wt% DTPA-K5 in DI water.

As DTPA-K5 being a salt (C_14_H_18_N_3_O_10_K_5_) and added as 5 wt% (50,000 ppm) the dielectric values differed with different water salinities, especially in the dielectric loss factor which is converted into conductivity. Measurements from 500 MHz to 1 GHz will be considered in the comparison. For all the measurements, adding DTPA-K5 to any brine will increase the conductivity (also increases Ɛ^″^ and decreases Ɛ_r_), which is caused by the increase of free ions due to the added salts. The change in the dielectric constant is more noticeable when the ionic ratio of added salts to the salinity of the brine is high. It can be noticed in the high difference of Ɛ_r_, Ɛ^″^ and conductivity in DI water whenever DTPA-K5 as 50,000 ppm (5 wt%) and 400,000 ppm (40 wt%) are added (Figs. 14 and 15). Also, in low salinity water the difference in dielectric parameters when adding DTPA-K5 (Figs. 12 and 13) is more than when added to seawater (Figs. 10 and 11). Which can be explained by, the frequency range of dielectric measurements is within 500 MHz to 1 GHz which is presenting the volumetric (bulk) polarization of the salts (or molecules) (Figs. 12, 13, 14, 15).

**Figure 16:**
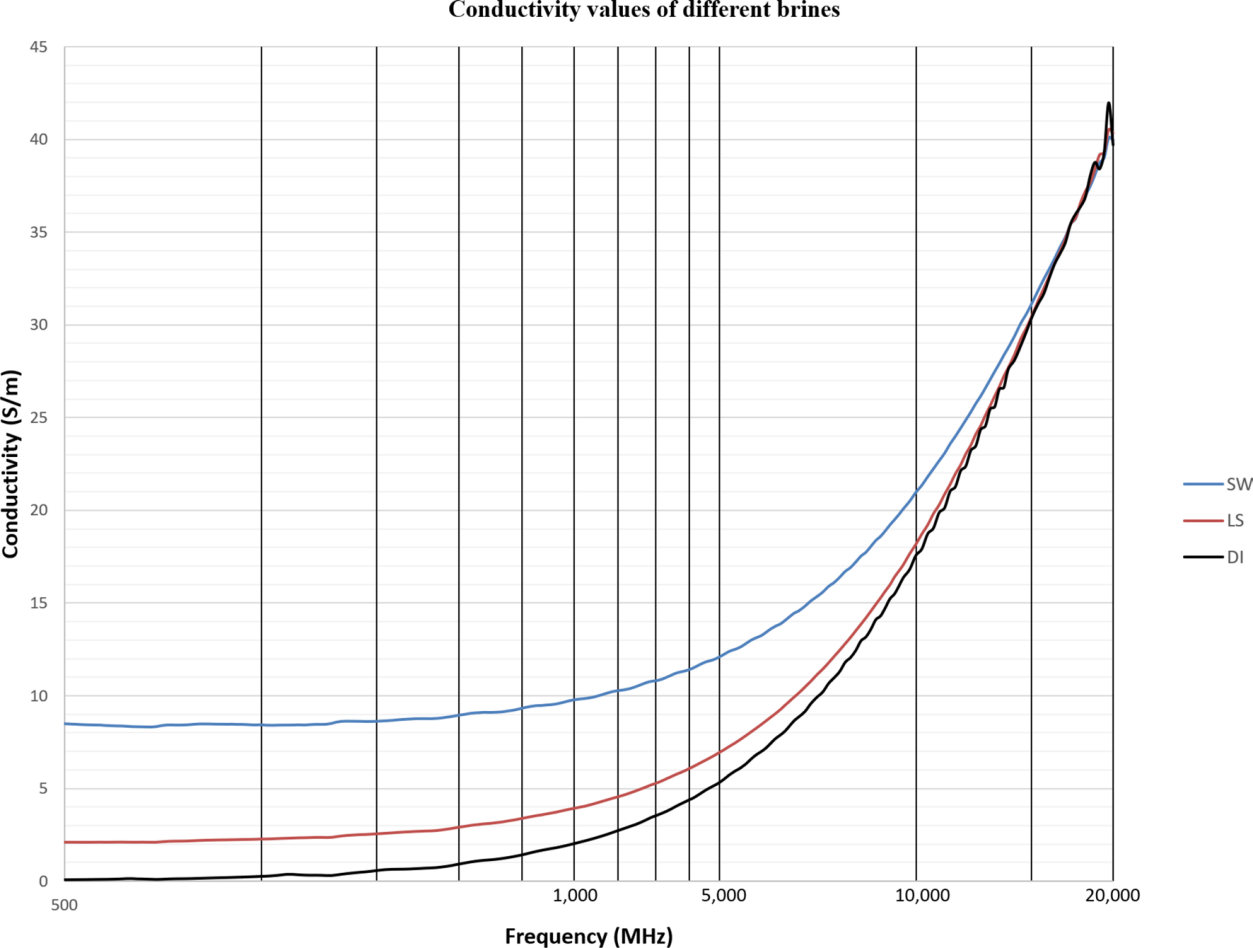
Conductivity ($\upsigma$) values of DI water, seawater and low salinity water.

**Figure 17:**
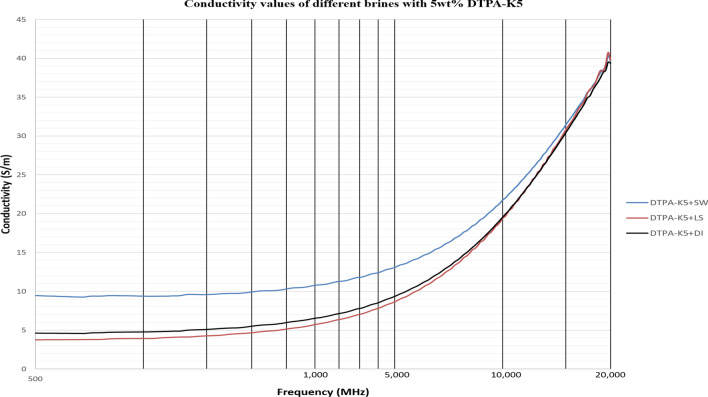
Conductivity ($\upsigma$) values of 5 wt% DTPA-K5 in DI water, 5 wt% DTPA-K5 in seawater and 5 wt% DTPA-K5 in low salinity water.

For all the measurements, the effect of ions presence is clear on the imaginary part of complex permittivity (meaning conductivity also) more than the real part (Ɛ_r_). As expected, the conductivity of a brine increases as the salinity increases (Fig. 16). Comparing the conductivity of seawater, low salinity water and DI water at frequency of 800 MHz are shown in Table 8. Adding DTPA-K5 to low salinity water showed lower conductivity than adding DTPA-K5 in DI water (Fig. 17)—which indicates less free ions due to the ion exchange occurring between the DTPA-K5 chelating agent (absorbed free cations) and the low salinity water cations. While the conductivity of seawater when DTPA-K5 is added is still higher than DTPA-K5 in DI or low salinity, where the chelating agent here have absorbed some of the free ions until it was fully saturated leaving behind free ions that increased the conductivity beyond low salinity water and DI water conductivity as shown in Fig. 17 and Table 8.

**Table 8:** Dielectric parameters of different brines at 800 MHz.

Liquid	Ɛ_r_	Ɛ^″^	Conductivity (S/m)
40wt% DTPA-K5 in DI	43.4	206.75	9.2
DI	79.3	3.09	0.14
5wt% DTPA-K5 in DI	72.3	105.7	4.7
SW	61.3	190.4	8.5
5wt% DTPA-K5 in SW	60.5	212	9.5
LS	76	49	2.177
5wt% DTPA-K5 in LS	75.3	87	3.88

### Effect of DTPA-K5 when added to different brines with different Fe^3+^ concentrations on the dielectric laboratory measurements

Different (Fe^3+^) concentrations are added (from 500 to 4000 ppm) according to Table 4 on Seawater, low salinity water and deionized water. The change on the dielectric parameters is recoded along with the change after adding 5wt% DTPA-K5 to these mixtures (brine with FeCl_3_ salt).

**Figure 18:**
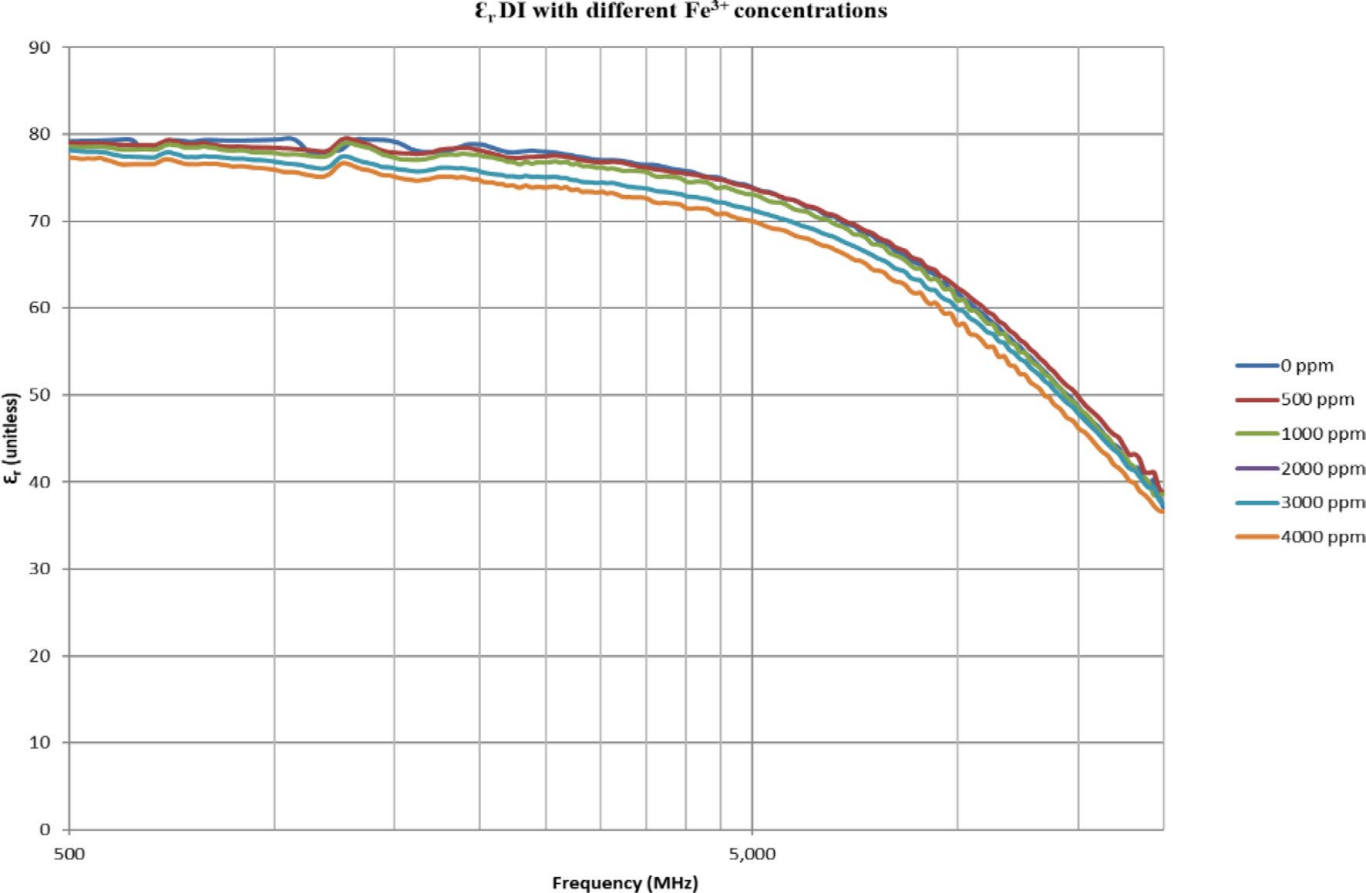
Relative dielectric constant (Ɛ_r_) values of DI water with different (Fe^3+^) concentrations.

**Figure 19:**
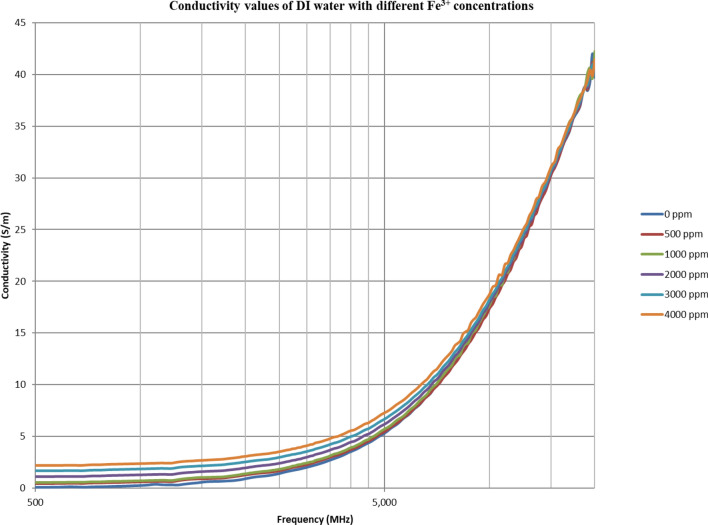
Conductivity ($\upsigma$) values of DI water with different (Fe^3+^) concentrations.

**Figure 20:**
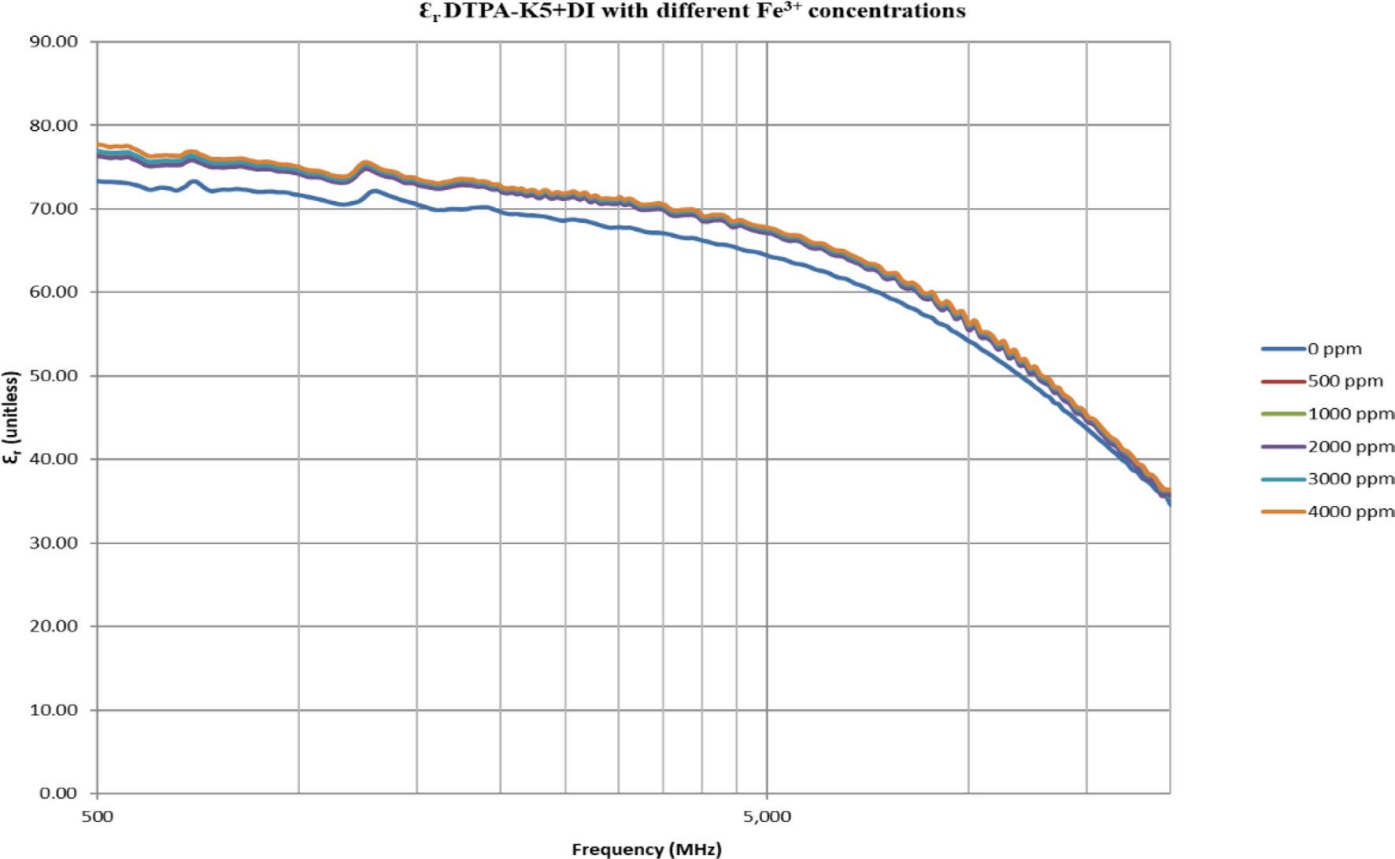
Relative dielectric constant (Ɛ_r_) values of 5 wt% DTPA-K5 in DI water with different (Fe^3+^) concentrations.

**Figure 21:**
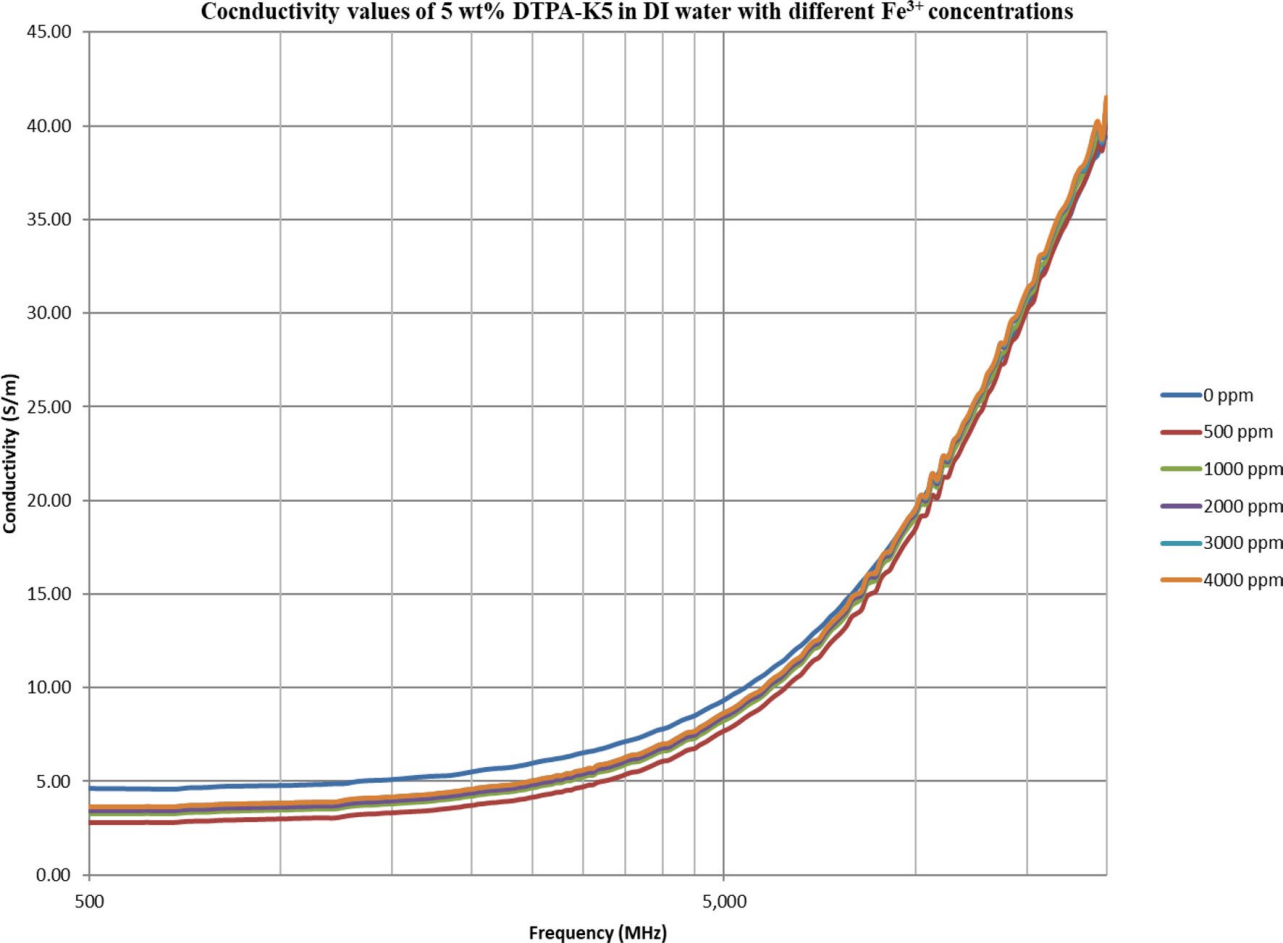
Conductivity ($\upsigma$) values of 5 wt% DTPA-K5 in DI water with different (Fe^3+^) concentrations.

**Figure 22:**
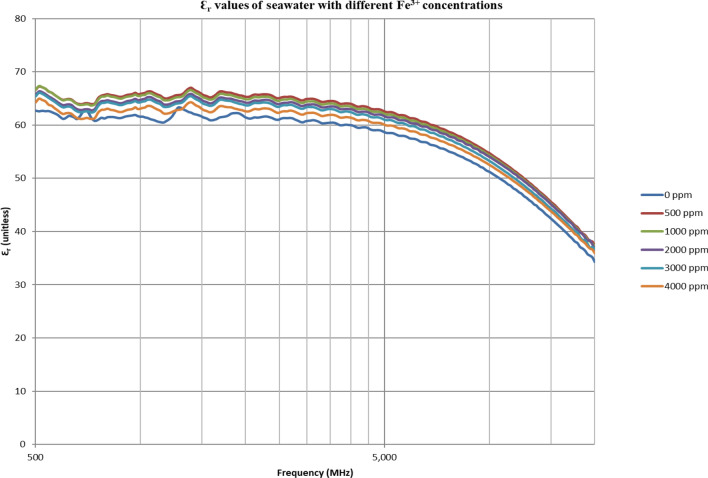
Relative dielectric constant (Ɛ_r_) values of seawater with different (Fe^3+^) concentrations.

**Figure 23:**
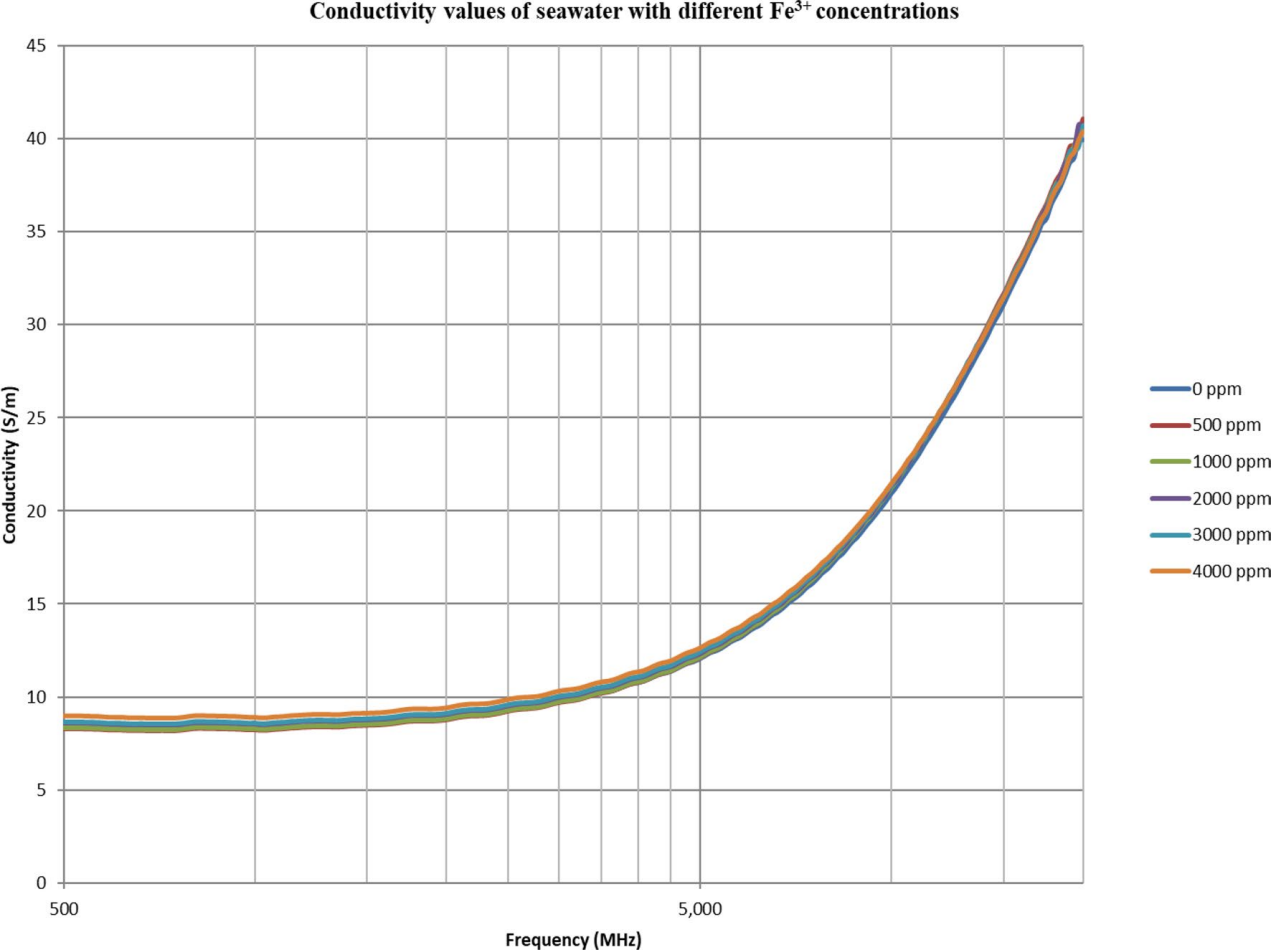
Conductivity ($\upsigma$) values of seawater with different (Fe^3+^) concentrations.

**Figure 24:**
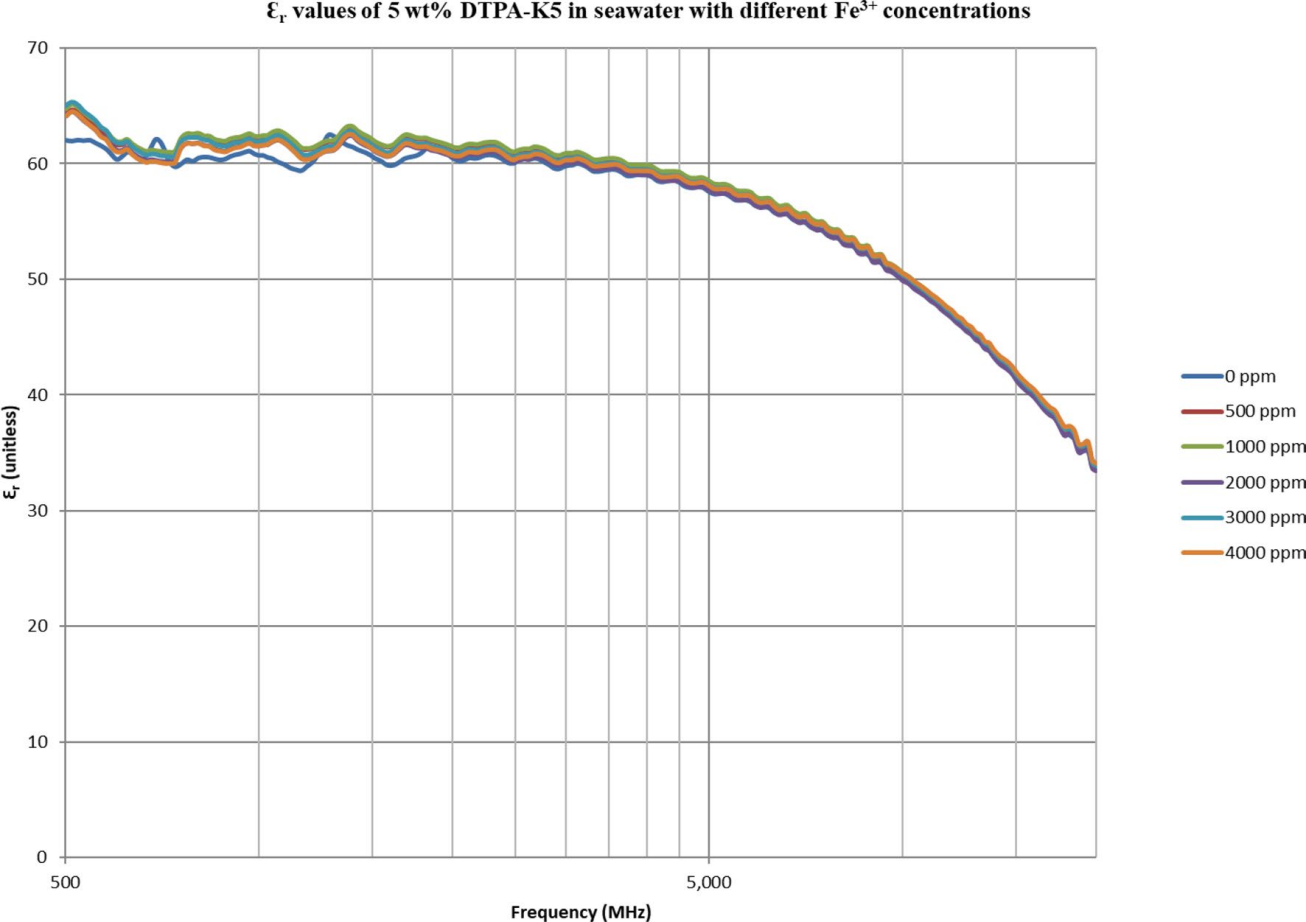
Relative dielectric constant (Ɛ_r_) values of 5 wt% DTPA-K5 in seawater with different (Fe^3+^) concentrations.

**Figure 25:**
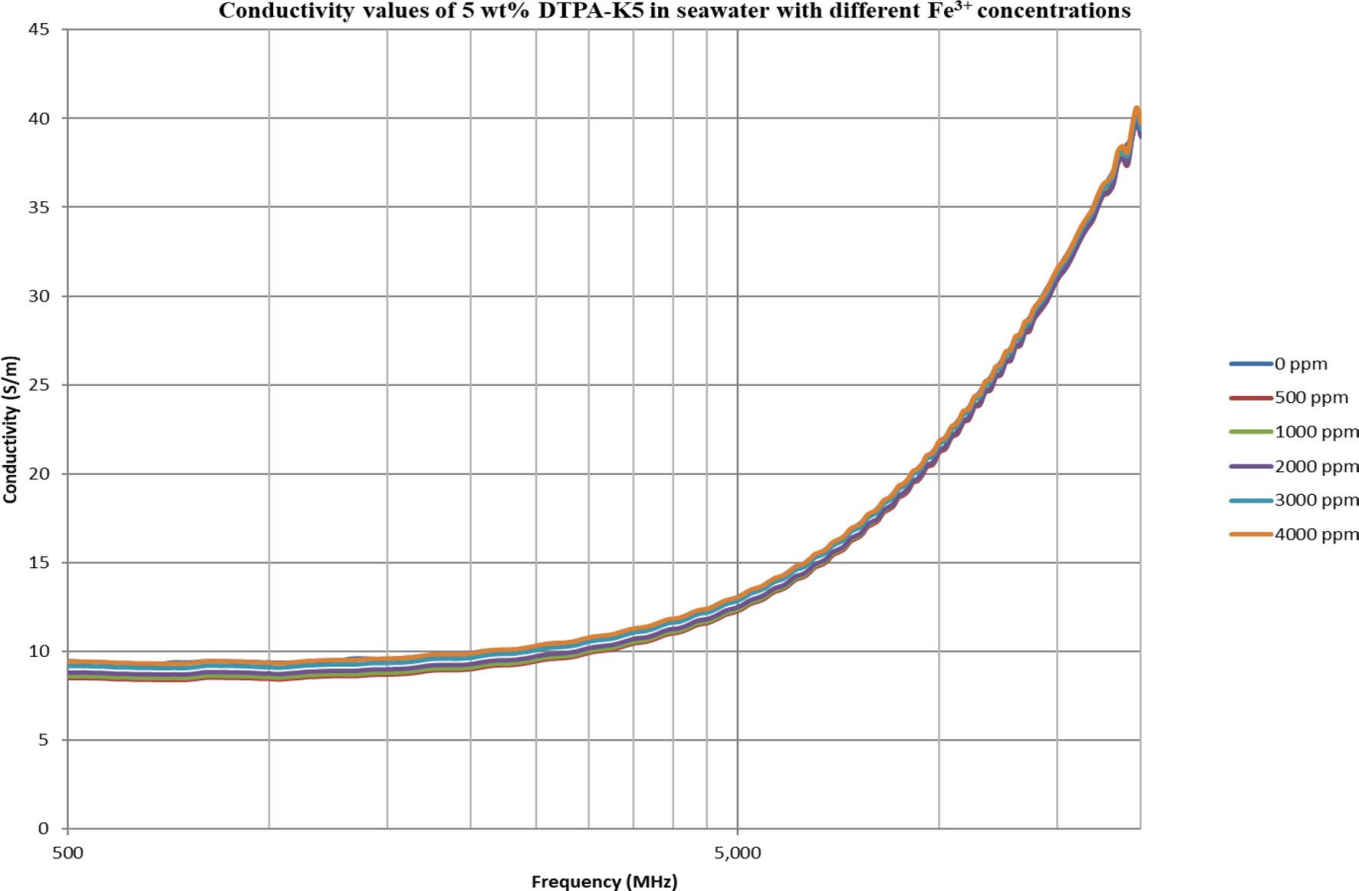
Conductivity ($\upsigma$) values of 5 wt% DTPA-K5 in seawater with different (Fe^3+^) concentrations.

**Figure 26:**
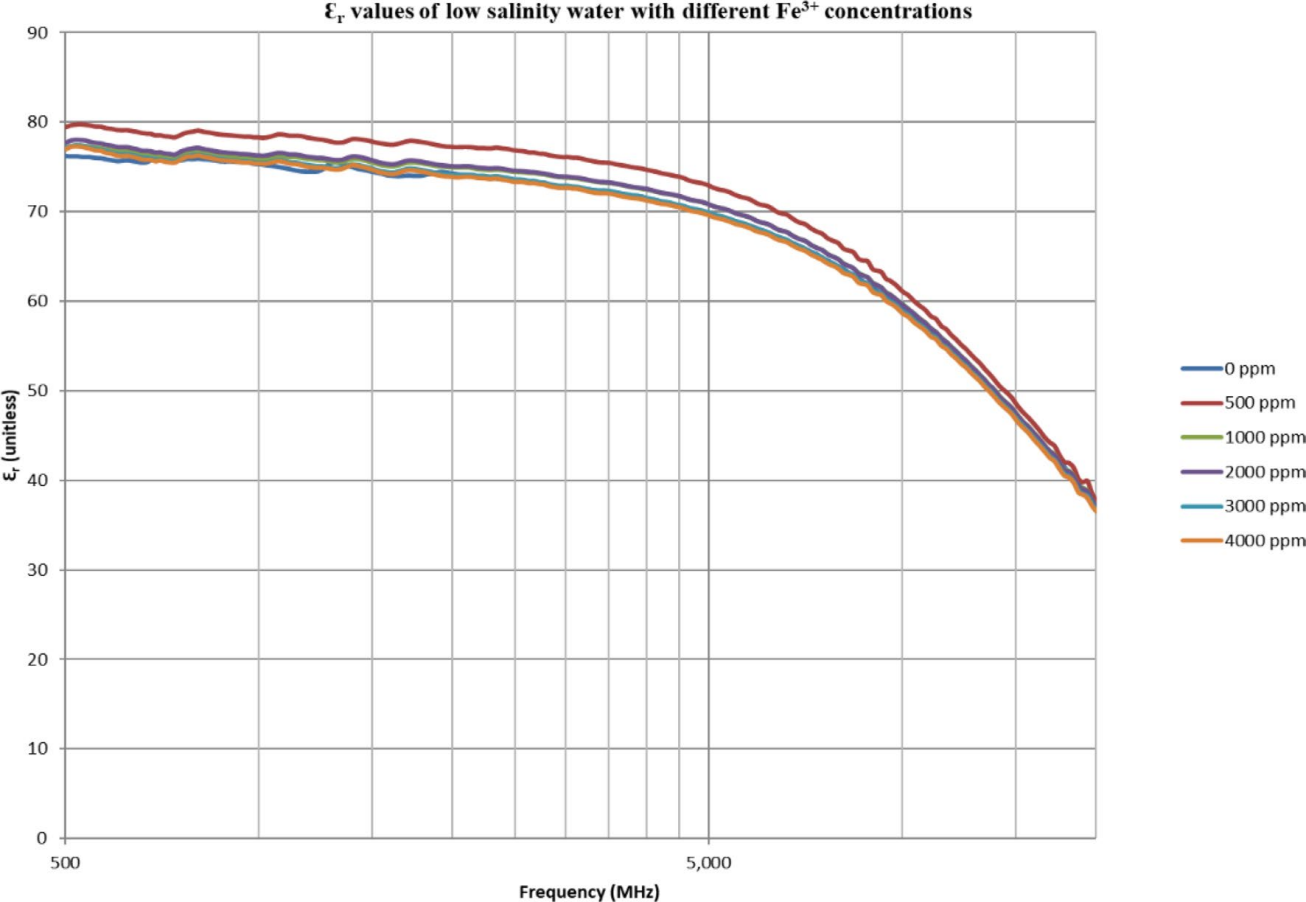
Relative dielectric constant (Ɛ_r_) values of low salinity water with different (Fe^3+^) concentrations.

**Figure 27:**
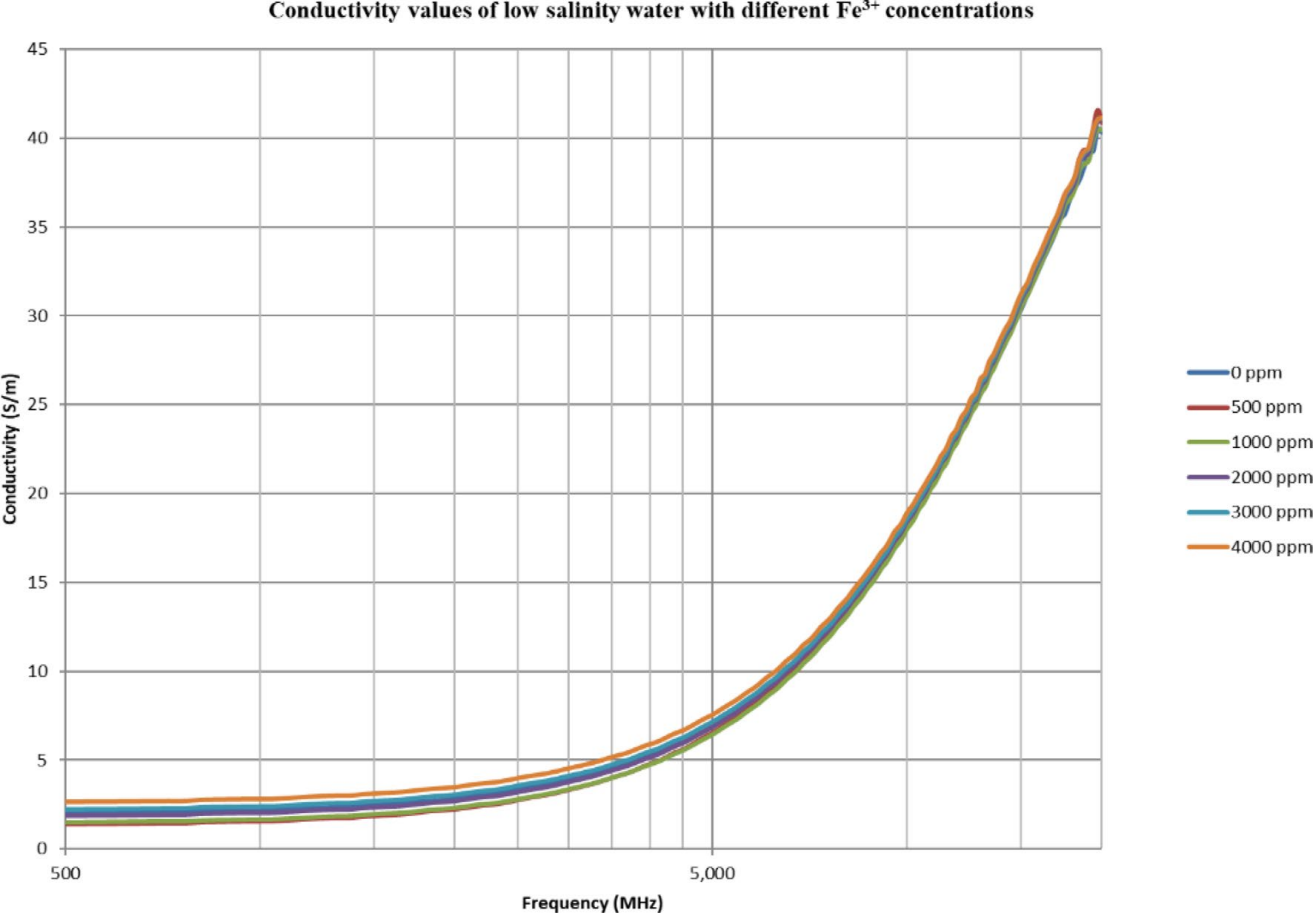
Conductivity ($\upsigma$) values of low salinity water with different (Fe^3+^) concentrations.

**Figure 28:**
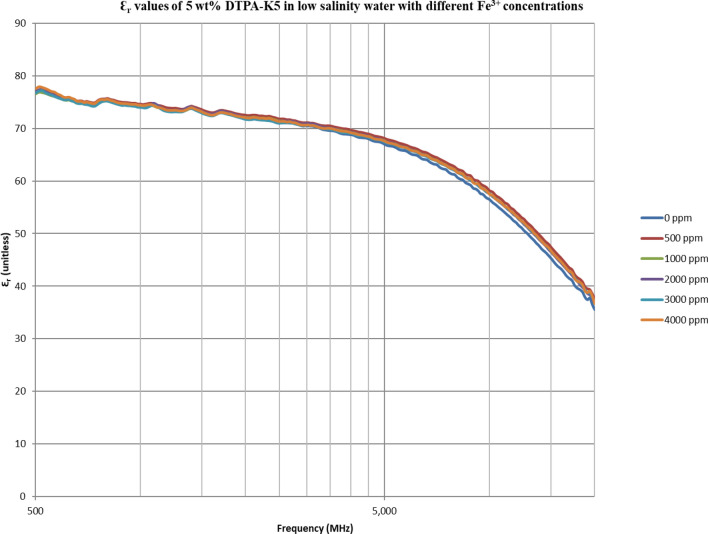
Relative dielectric constant (Ɛ_r_) values of 5 wt% DTPA-K5 in low salinity water with different (Fe^3+^) concentrations.

**Figure 29:**
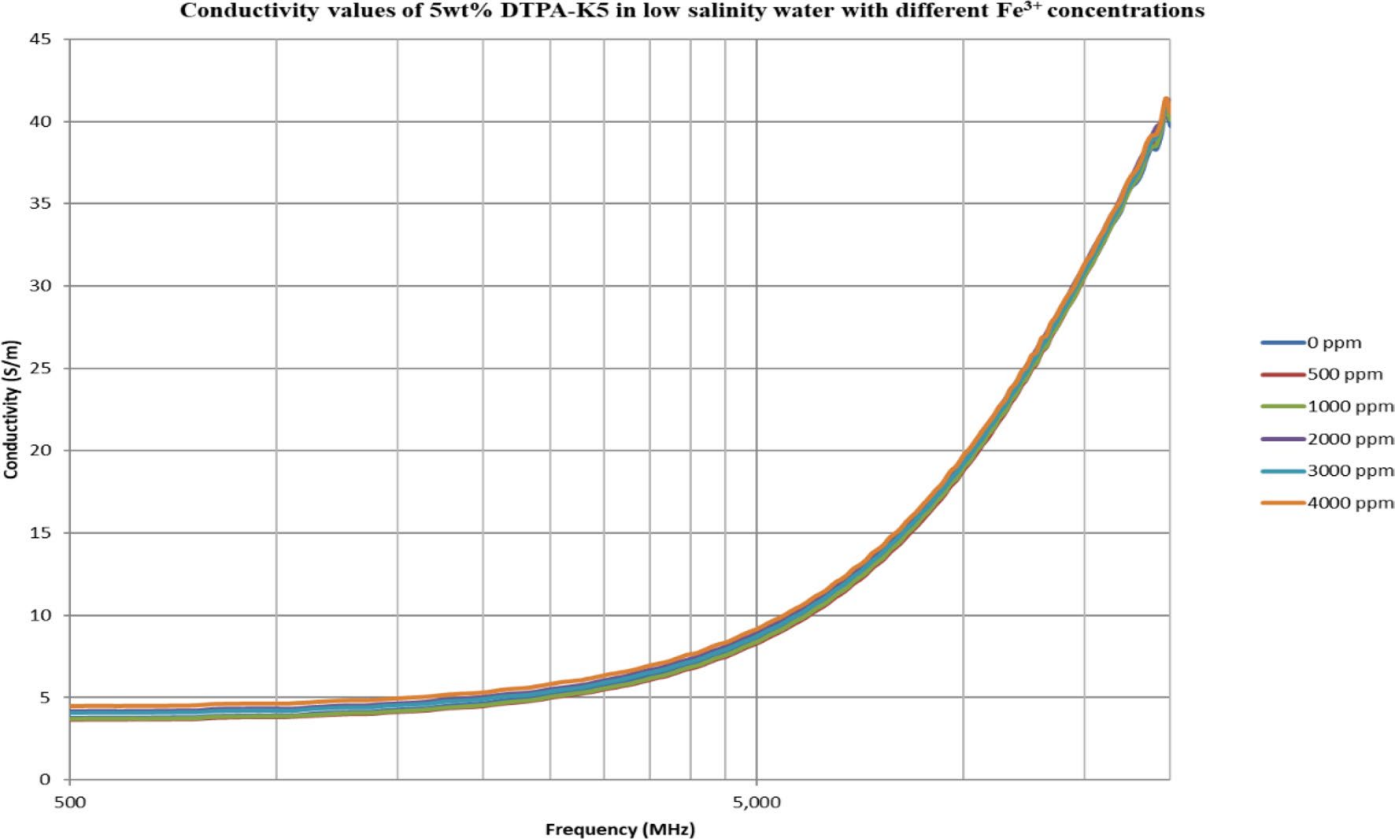
Conductivity ($\upsigma$) values of 5 wt% DTPA-K5 in low salinity water with different (Fe^3+^) concentrations.

**Figure 30:**
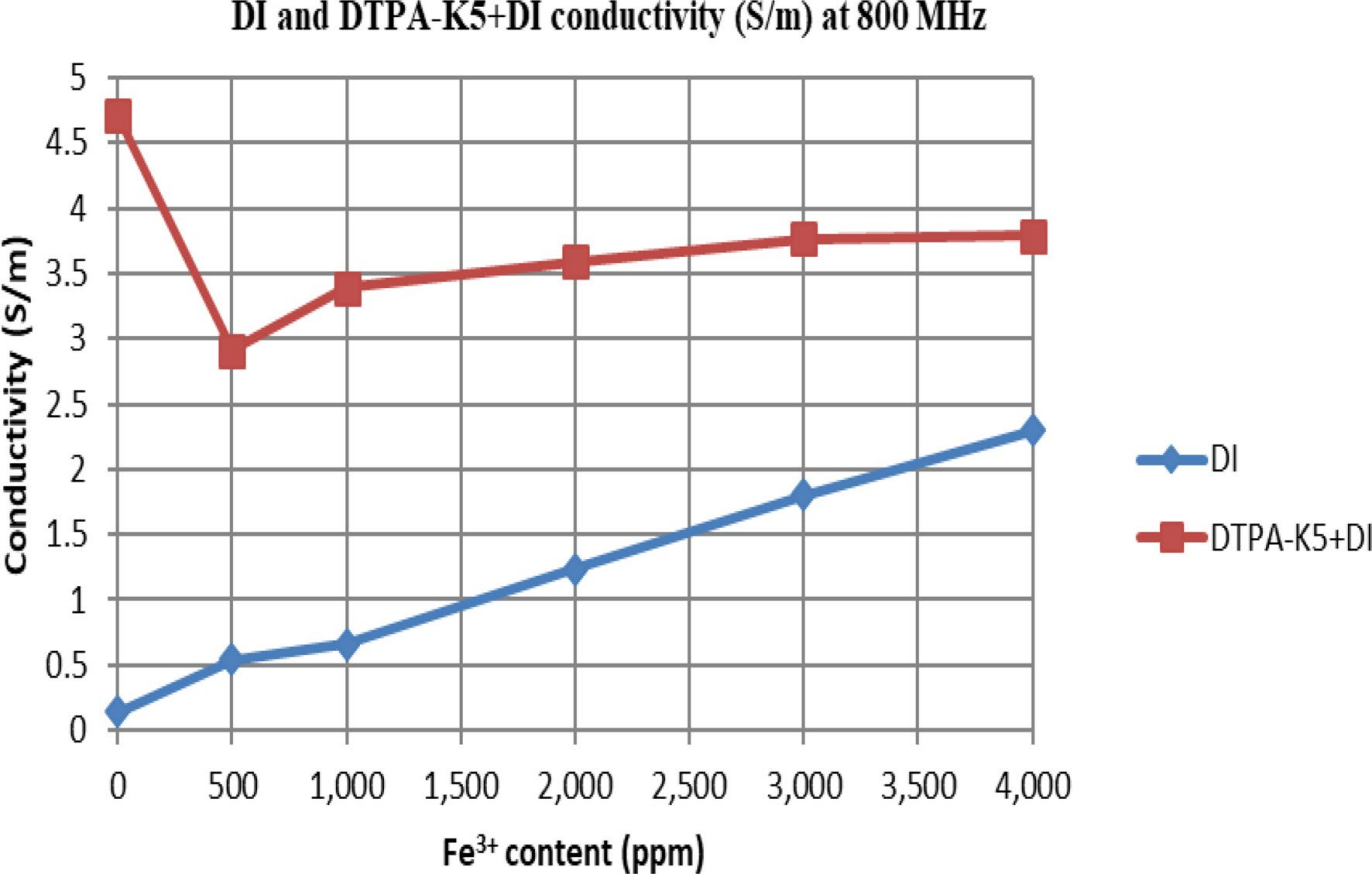
Conductivity ($\upsigma$) values at 800 MHz of DI water and 5 wt% DTPA-K5 in DI water with different (Fe^3+^) concentrations.

**Figure 31:**
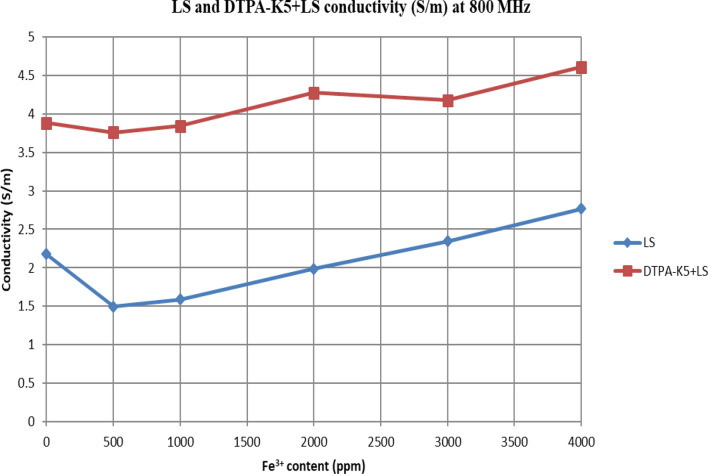
Conductivity ($\upsigma$) values at 800 MHz of low salinity water and 5 wt% DTPA-K5 in Low salinity water with different (Fe^3+^) concentrations.

**Figure 32:**
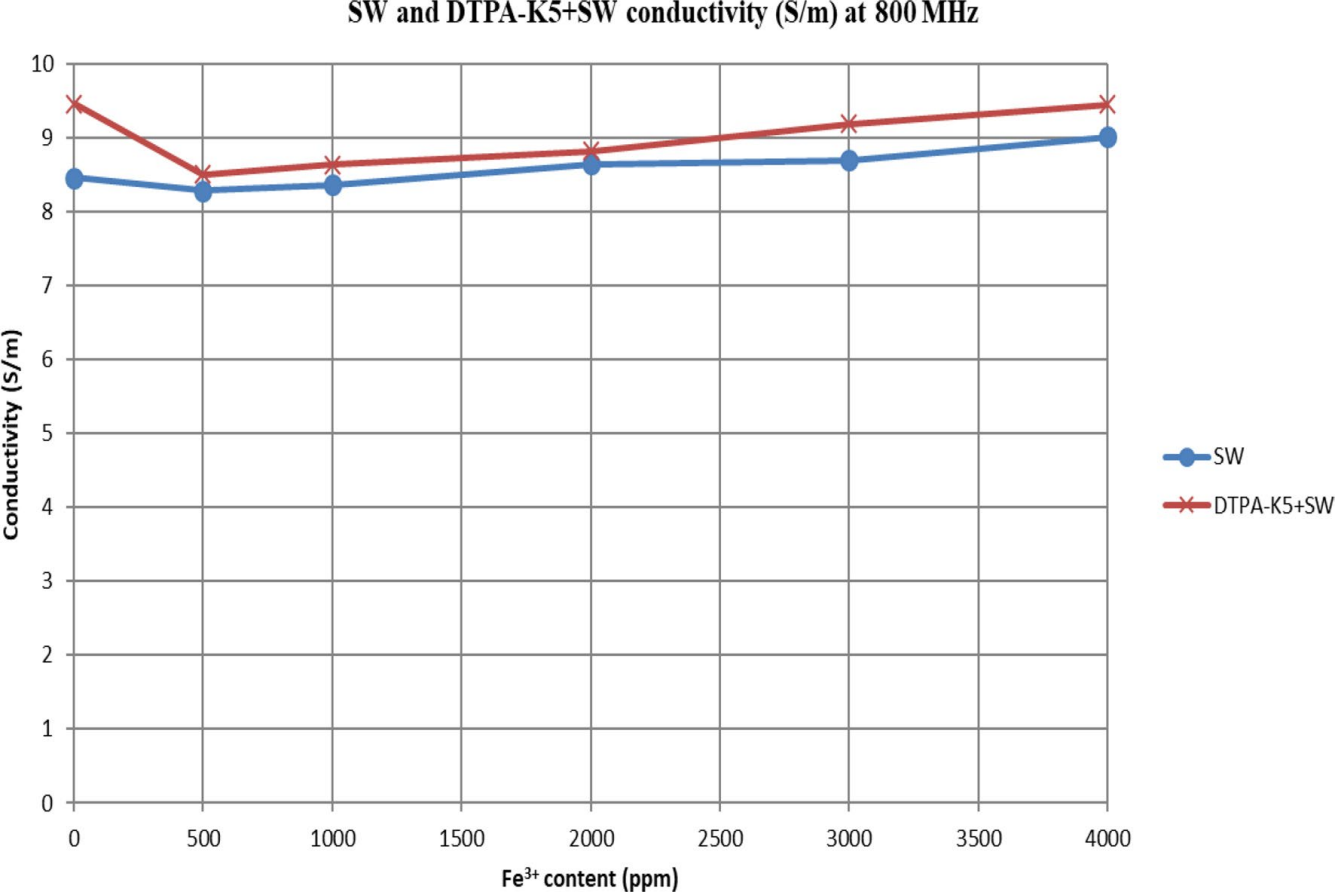
Conductivity ($\upsigma$) values at 800 MHz of seawater and 5 wt% DTPA-K5 in seawater with different (Fe^3+^) concentrations.

From Figs. 18, 20, 22, 24, 26 and 28, the change in the real (relative) dielectric constant (Ɛ_r_) as ferric ion concentrations increases is not noticeable and lies within the device’s uncertainty. The effect of adding FeCl_3_ salt is much clearer when added to DI water then when added to low salinity water while its effect when added to seawater is the lowest (Figs. 18, 19, 20, 21, 22, 23, 24, 25, 26, 27, 28, 29). Ignoring the change on the real part of dielectric permittivity (since it lies with the device’s uncertainty) a summary of the change on conductivity at 800 MHz are plotted (Figs. 30, 31, 32). In Fig. 30, a comparison between adding ferric ion to DI water and to 5 wt% DTPA-K5 in DI water is plotted. FeCl3 salt is added gradually to both DI water and DTPA + DI water mixtures. When FeCl3 salt is added to DI water, a continuous increase in conductivity is observed. With 500 ppm Fe^3+^ added to DI water, conductivity naturally increased to around 0.5 S/m. On the other hand, when the 500 ppm Fe^3+^ are added to DTPA + DI, a decrease of 38% in the mixture’s conductivity is observed (from 4.7 to 2.9 S/m). Furthermore, with the increase in Fe^3+^ concentration, the conductivity value starts to increase but in a slower rate than the increase observed in DI water when Fe^3+^ concentration went from 500 to 4000 ppm. The rate of increase of the conductivity when adding 500 ppm to 4000 ppm Fe^3+^ in DTPA + DI water is 0.025%, while in DI water the rate of increase in conductivity is 0.05%.

In Figs. 31 and 32, both brines (seawater and low salinity water) experience a decrease in the conductivity when 500 ppm of Fe^3+^ is added (the decrease in low salinity is higher—equals 31.21%- than the decrease in seawater—equals 2.16%). Then, the conductivity increases as Fe^3+^ concentrations increases. Also, whenever 5 wt% DTPA-K5 is added the conductivity decreases when 500 ppm Fe^3+^ is added then starts to increase as Fe^3+^ concentration increases. Adding 500 ppm of Fe^3+^ to Low salinity water will lead to reduction on the low salinity water conductivity (31.2% decrease)—less free ions—but it is a much more reduction on low salinity water conductivity than: Adding DTPA-K5 and 500 ppm of Fe^3+^ to low salinity water (3.22% decrease) (Fig. 31). The effect on conductivity of adding DTPA-K5 in seawater starts to be negligible after adding 1000 ppm Fe^3+^ (Fig. 32). Also, adding 500 ppm of Fe^3+^ to seawater will lead to reduction on the seawater conductivity (2.1% decrease)—less free ions—but it is a less reduction on seawater conductivity than: Adding DTPA-K5 and 500 ppm of Fe^3+^ to seawater (10% decrease).

### Effect on conductivity at high frequency of adding DTPA-K5 to deionized water with different salts

**Figure 33:**
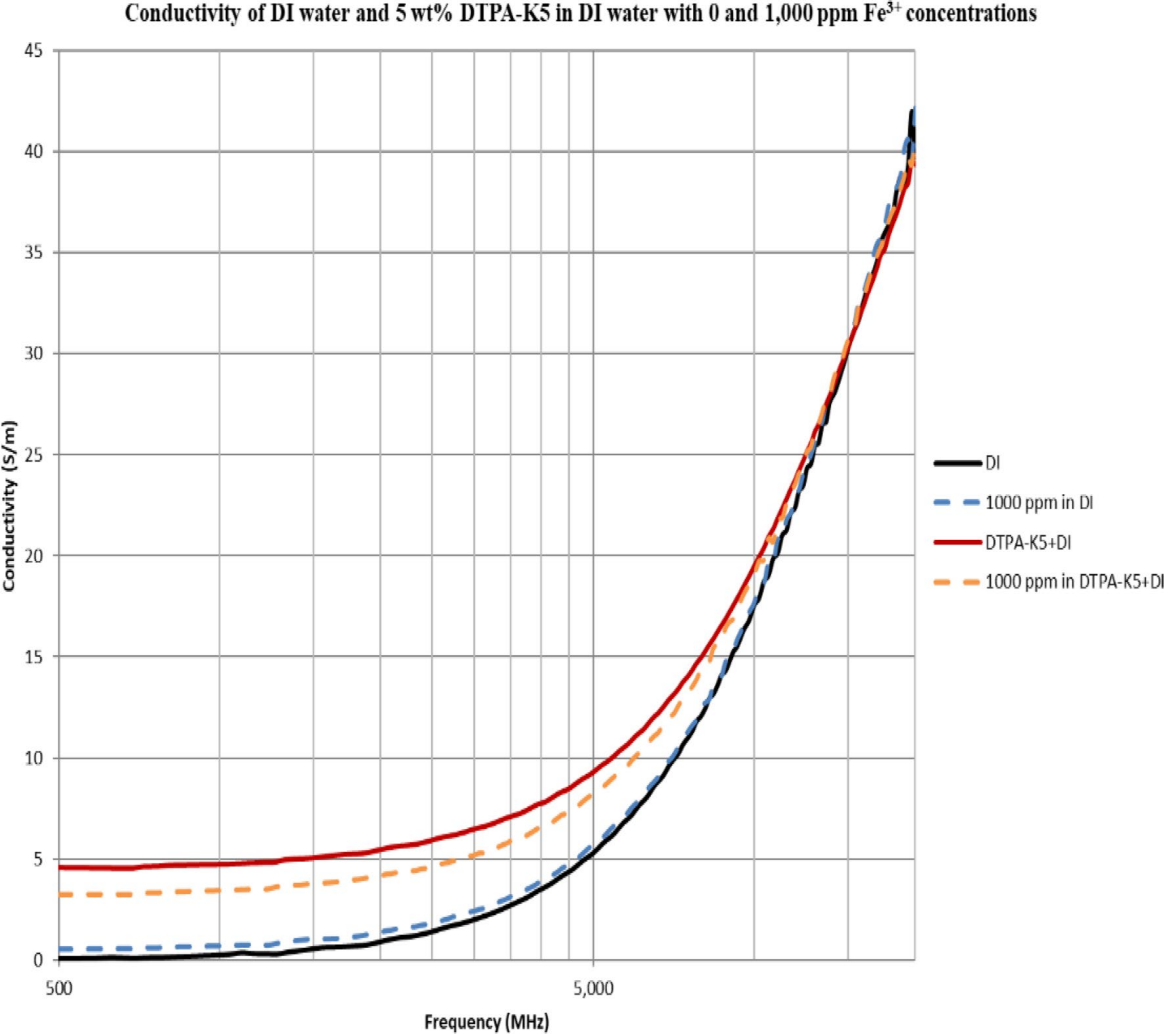
Conductivity ($\upsigma$) values of DI water and 5 wt% DTPA-K5 in DI water with 0 and 1000 ppm (Fe^3+^) concentrations.

**Figure 34:**
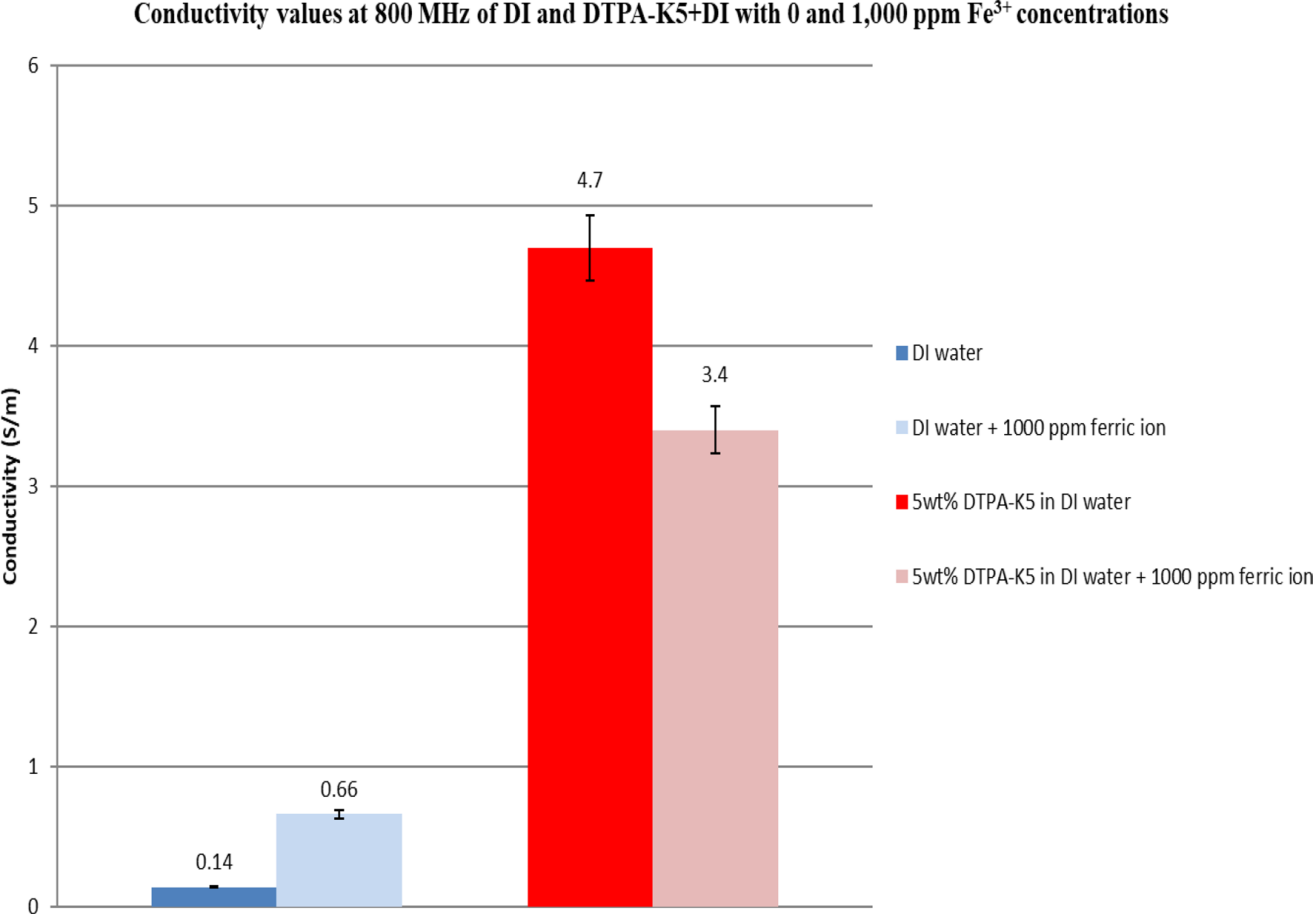
Comparison between the conductivity ($\upsigma$) values at 800 MHz of DI water and 5 wt% DTPA-K5 in DI water with 0 and 1000 ppm (Fe^3+^) concentrations.

**Figure 35:**
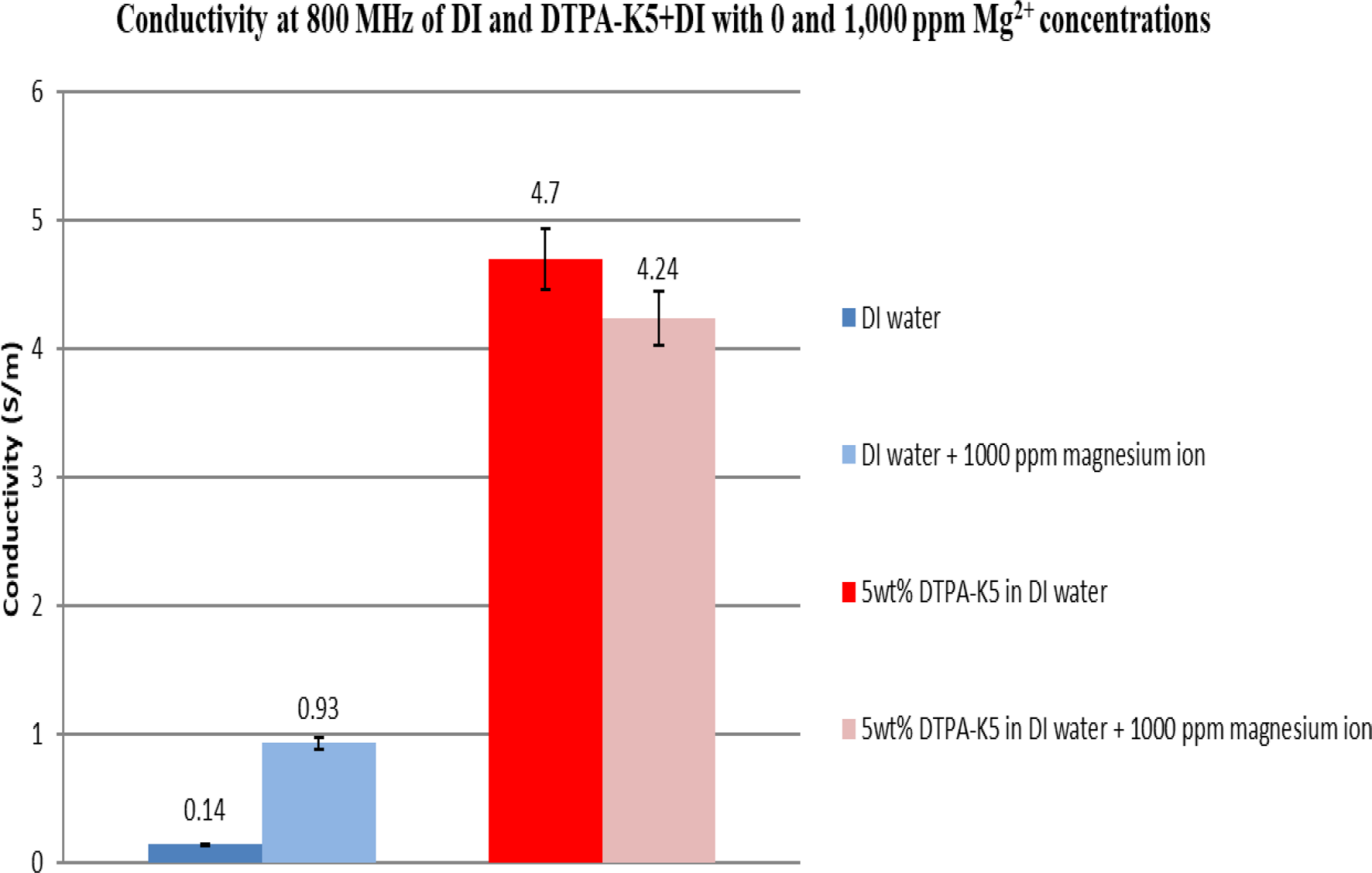
Comparison between the conductivity ($\upsigma$) values at 800 MHz of DI water and 5 wt% DTPA-K5 in DI water with 0 and 1000 ppm (Mg^2+^) concentrations.

**Figure 36:**
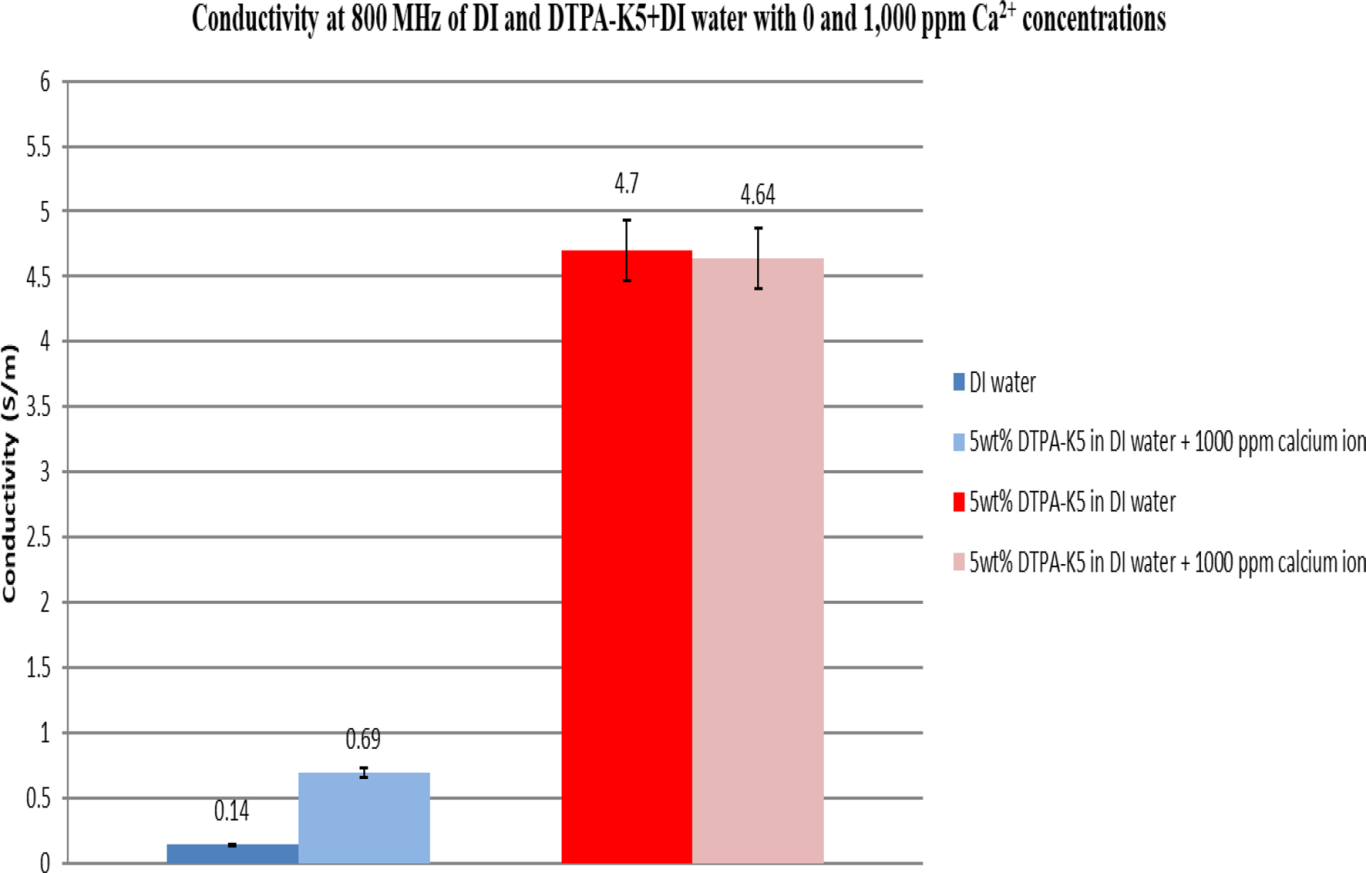
Comparison between the conductivity ($\upsigma$) values at 800 MHz of DI water and 5 wt% DTPA-K5 in DI water with 0 and 1000 ppm (Ca^2+^) concentrations.

**Figure 37:**
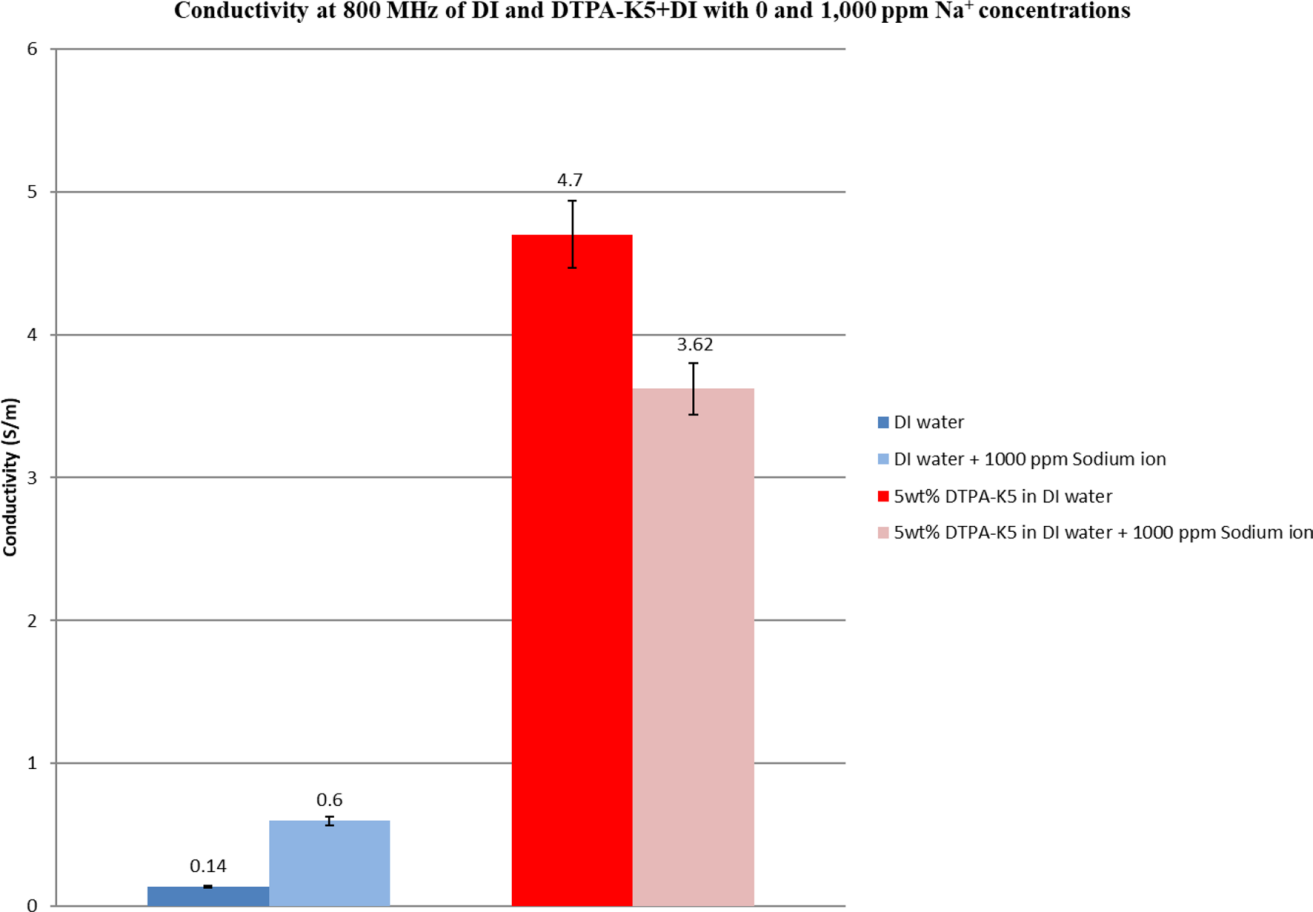
Comparison between the conductivity ($\upsigma$) values at 800 MHz of DI water and 5 wt% DTPA-K5 in DI water with 0 and 1000 ppm (Na^+^) concentrations.

Adding the multivalent (Fe^3+^, Mg^2+^ and Ca^2+^) and monovalent (Na^+^) cations to DI water will increase the conductivity. Also, adding DTPA-K5 salt with concentration of 50,000 ppm (5 wt%) will also increase the conductivity of DI water. But, when adding any of the FeCl_3_, MgCl_2_, CaCl_2_ or NaCl salts containing each 1000 ppm of the Fe^3+^, Mg^2+^, Ca^2+^ and Na^+^ cations, respectively (following the calculations in Tables Tables 4–7), to DI water with DTPA-K5, the mixture conductivity decreases (Figs. 33, 34, 35, 36, 37). For a frequency ranging from 500 MHz to 1 GHz, the reduction in conductivity is observed when 1000 ppm of Fe^3+^ is added to the DTPA-K5 with DI mixture while conductivity increased naturally when the same amount is added to DI water (Fig. 33). For the divalent elements (Mg^2+^ and Ca^2+^) the reduction in the DI with DTPA-K5 is less (Figs. 35 and 36) than the reduction occurs when adding the trivalent or monovalent elements (Fe^3+^ and Na^+^ respectively) (Figs. 34 and 37) at 800 MHz frequency. The addition of salts to DI water increases the conductivity as expected due to the presence of the free ions from the salts. On the contrary, whenever DTPA-K5 chelating agent is present in DI water, the addition of salts to the water will reduce the conductivity rather than increasing it. This is believed to be due the absorption of cations by the chelating agent resulting in a reduction of the free cations on the liquid (less conductive liquid). Furthermore, it can be interpreted from the conductivity results in the presence of DTPA-K5 with the different cations that the higher the reduction in conductivity after the addition of the cations could indicate a higher absorption potential by DTPA-K5 of the added cations.

Based on the results obtained from the laboratory dielectric experiments, the presence of DTPA-K5 resulted in chelating the free cations (multivalent and monovalent) which is reflected by the decrease in conductivity after adding the salts. This is an indication that the metal ions (Fe^3+^, Mg^2+^, Ca^2+^ and Na^+^) have been totally sequestered by the chelating agent reducing the conductivity of the mixture due to less free cations form DTPA-K5 and the added salts.

For the utilization of the dielectric laboratory test, it can be used as an evaluation technique into the ion exchange that occurs between different fluids from the reservoir with different brines and additives. Also, it can be used to determine the maximum chelating capacity of different chelating agents with different cations which is reflected by the change in conductivity.

## Conclusions

Through comprehensive study on conductivity and dielectric measurements on different brines, a chelating agent and sandstone rocks, it was observed that:

- The frequency range of dielectric measurements is with high frequency (500 MHz to 1 GHz), presenting the volumetric (bulk) polarization of the salts (molecules).
- The effect of ion exchange is clear on the imaginary part of complex permittivity (high frequency conductivity) rather than the real dielectric constants.
- The double layer effect is not observed using the dielectric measurements due to the high frequency which bypasses the double layer effect.
- Adding salts did not lead to increase in conductivity in the presence of DTPA-K5 chelating agent. Conductivity decreases due to the absorption of free ions by DTPA-K5.
- Adding DTPA-K5 to low salinity water led to lower conductivity at high frequency than DTPA-K5 added to DI water, which shows less free ions due to the ion exchange occurred.
- The laboratory dielectric measurement tool could be a valuable tool in the lab to study the fluid–fluid interaction activities. It can be used as an evaluation technique into the ion exchange that occurs between different fluids from the reservoir with different brines and additives. Also, it can be utilized to investigate the maximum chelating capacity of different chelating agents with different cations.

## Data Availability

The datasets used and/or analyzed during the current study available from the corresponding author on reasonable request.

## List of symbols

DI: Deionized water
DTPA-K5: Diethylenetriaminepentaacetic acid, potassium salt
Ɛ^*^: Complex permittivity, unitless
Ɛ_r_ or Ɛ^′^: Relative dielectric constant (relative permittivity), unitless
Ɛ_0_: Vacuum permittivity, F/m
Ɛ^″^: Dielectric loss factor, unitless
ƒ: Frequency, 1/second or Hertz
LS: Low salinity water
ppm: Parts per million
SW: Seawater
$$\sigma $$: Conductivity, S/m
$\omega$: Circular frequency, rd/s
