# Author Correction: Laboratory dielectric measurements to evaluate the conductivity change in the presence of chelating agent with different brines

**DOI:** 10.1038/s41598-023-33555-8

**Published:** 2023-05-01

**Authors:** Sulaiman A. Alarifi, Mohamed Mahmoud

**Affiliations:** Department of Petroleum Engineering, King Fahd University of Petroleum & Minerals (KFUPM), Dhahran, 31261 Saudi Arabia

Correction to: *Scientific Reports* https://doi.org/10.1038/s41598-022-23964-6, published online 18 November 2022

The original version of this Article contained errors.

Salah Al-Ofi was incorrectly listed as an author of the original Article, and has subsequently been removed.

The Author Contribution section now reads:

“S.A. and M.M. contributed to conception and planning of the study. S.A. and M.M. organized and supervised the work. S.A. performed the experiments. S.A. performed data analysis and representation. S.A. wrote the first draft of the manuscript. All authors contributed to manuscript revision, read, and approved the submitted version.”

Additionally, the Acknowledgement section:

“The authors of this paper gratefully acknowledge the support provided by the department of Petroleum Engineering at King Fahd University of Petroleum & Minerals (KFUPM).”

now reads:

“The authors express their sincere gratitude to Schlumberger Dhahran Center for Carbonate Research (SDCR) for providing the necessary facilities and equipment to conduct the laboratory experiments featured in this study. This work was carried out in collaboration with the Department of Petroleum Engineering at King Fahd University of Petroleum & Minerals (KFUPM). The authors would like to acknowledge Mr. Salah Al-Ofi for his help and support.”

The original Article has been corrected.

